# EPHA2 is a novel cell surface marker of OCT4-positive undifferentiated cells during the differentiation of mouse and human pluripotent stem cells

**DOI:** 10.1093/stcltm/szae036

**Published:** 2024-05-29

**Authors:** Atsushi Intoh, Kanako Watanabe-Susaki, Taku Kato, Hibiki Kiritani, Akira Kurisaki

**Affiliations:** Division of Biological Science, Nara Institute of Science and Technology, Nara, 630-0192, Japan; Organ Development Research Laboratory, National Institute of Advanced Industrial Science and Technology (AIST), Tsukuba, 305-8560, Japan; Organ Development Research Laboratory, National Institute of Advanced Industrial Science and Technology (AIST), Tsukuba, 305-8560, Japan; Division of Biological Science, Nara Institute of Science and Technology, Nara, 630-0192, Japan; Division of Biological Science, Nara Institute of Science and Technology, Nara, 630-0192, Japan; Division of Biological Science, Nara Institute of Science and Technology, Nara, 630-0192, Japan; Organ Development Research Laboratory, National Institute of Advanced Industrial Science and Technology (AIST), Tsukuba, 305-8560, Japan

**Keywords:** EPHA2, pluripotent stem cells, cell surface marker, tumorigenesis, stem cell transplantation

## Abstract

Embryonic stem cells (ESCs) and induced pluripotent stem cells (iPSCs) possess the intrinsic ability to differentiate into diverse cellular lineages, marking them as potent instruments in regenerative medicine. Nonetheless, the proclivity of these stem cells to generate teratomas post-transplantation presents a formidable obstacle to their therapeutic utility. In previous studies, we identified an array of cell surface proteins specifically expressed in the pluripotent state, as revealed through proteomic analysis. Here we focused on EPHA2, a protein found to be abundantly present on the surface of undifferentiated mouse ESCs and is diminished upon differentiation. Knock-down of *Epha2* led to the spontaneous differentiation of mouse ESCs, underscoring a pivotal role of EPHA2 in maintaining an undifferentiated cell state. Further investigations revealed a strong correlation between EPHA2 and OCT4 expression during the differentiation of both mouse and human PSCs. Notably, removing EPHA2^+^ cells from mouse ESC-derived hepatic lineage reduced tumor formation after transplanting them into immune-deficient mice. Similarly, in human iPSCs, a larger proportion of EPHA2^+^ cells correlated with higher OCT4 expression, reflecting the pattern observed in mouse ESCs. Conclusively, EPHA2 emerges as a potential marker for selecting undifferentiated stem cells, providing a valuable method to decrease tumorigenesis risks after stem-cell transplantation in regenerative treatments.

## Graphical Abstract

**Graphical Abstract:**
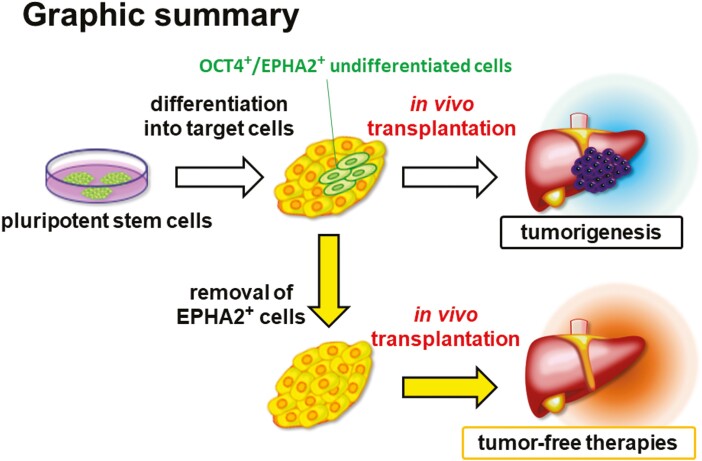

**Significance statement**

Pluripotent stem cells hold remarkable promise for regenerative medicine, given their strong differentiation ability into various cell types. However, their propensity to form teratomas highlights the critical need for strategies to eliminate tumorigenic cells from therapeutic preparations. We found EPHA2 as a transmembrane protein that co-expresses with OCT4 in pluripotent stem cell-derived populations. Significantly, EPHA2 can be used as an efficient cell surface marker in distinguishing tumorigenic undifferentiated cells from their differentiated counterparts. These findings present advancements that could bridge the gap between stem cell research and clinical application.

## Introduction

EPHA2, a receptor tyrosine kinase, is activated during early development and pathogenesis. It plays a crucial role in managing a range of biological functions, including cell proliferation and migration.^1^ In the context of cancer, EPHA2 serves as one of the primary functional receptors among EPH type A members and is implicated in the enhancement of tumorigenesis by modulating cell-cell interactions, proliferation,^2^ and cancer cell motility.^3^ These cellular actions are mediated by intricate signaling pathways like KRAS activation and ERK inhibition.^4,5^ Consequently, EPHA2 is posited as a significant cell surface marker for identifying and tracking the progression of aggressive and malignant tumors.

Pluripotent stem cells (PSCs), including embryonic stem cells (ESCs) and induced pluripotent stem cells (iPSCs), are renowned for their self-renewal and diverse differentiation capabilities. These characteristics render PSCs promising for regenerative medicine applications. However, their tendency to develop teratomas after transplantation in vivo^6-8^ emphasizes the need to remove tumorigenic undifferentiated stem cells to reduce the risk of unintended tumor formation.

Existing markers like TRA-1-60, TRA-1-81,^9^ SSEAs,^10^ and L1CAM^11^ have been instrumental but are not wholly effective in isolating these cells. This underscores the need for additional specific cell surface markers to enhance the safety of stem-cell transplantation. Our prior work identified several proteins, including EPHA2, on the plasma membrane of undifferentiated mouse ESCs,^12^ but the comprehensive role of EPHA2 in pluripotency and as a marker for tumorigenic cells is still not fully understood.

In this study, we show that EPHA2 plays a crucial role in regulating pluripotency. We found that EPHA2, a transmembrane protein, is co-expressed with OCT4 in mouse and human PSCs. Our in vivo transplantation experiments also indicate that EPHA2 serves as a useful cell surface marker for separating tumorigenic undifferentiated cells from differentiated ones. These findings offer novel perspectives on the roles of EPHA2 in pluripotency, differentiation, and tumorigenesis, offering avenues for enhanced safety in stem-cell transplantation.

## Materials and methods

### Cell culture

The mouse embryonic stem cell (ESC) line D3 was cultured with Dulbecco’s Modified Eagle Medium (DMEM, High-Glucose, Nacalai Tesque) enriched with a 15% ES-qualified, heat-inactivated fetal bovine albumin (FBS, Gibco-BRL), 0.1 mM β-mercaptoethanol, 1 × non-essential amino acids (NEAA, Nacalai Tesque), 100 units/mL penicillin and 100 μg/mL streptomycin (1 × P/S, Nacalai Tesque). This ES growth medium was further supplemented with 1000 units/mL LIF or 2i (3 μM CHIR99021, CAYMAN, and 1 μM PD0325901, Selleckchem).^13^ The cells were cultured on dishes coated with 0.1% gelatin to facilitate optimal cell adhesion. The E14 and B6G2 mouse ESC lines were cultured on mitomycin C-treated mouse embryonic fibroblasts (MEFs). All cells were cultured in a humidified incubator, maintaining an atmosphere of 5% CO_2_ at a stable temperature of 37 °C.

### Differentiation of mouse ESCs

Before differentiation, mouse ESCs, E14, and B6G2, cultured on MEFs were plated on a gelatin-coated dish for 30 minutes to remove MEFs. The floating ESCs were harvested and cultured in a LIF-free ES growth medium to initiate spontaneous differentiation. For directed differentiation toward hepatic cell lineages, the ESCs were trypsinized and transferred to a 96-well low-binding plate (Thermo Fisher Scientific) to facilitate the formation of embryoid bodies (EBs) following the previously reported methodologies.^14,15^ Briefly, the EBs were cultured in a blend of 75% Iscove’s Modified Dulbecco’s Medium (IMDM, Cellgro) and 25% Ham’s F12 medium (Cellgro) supplemented with 0.5 × N2, 0.5 × B27 (Gibco-BRL), 1 × P/S, 0.05% bovine serum albumin, 2 mM glutamine (Gibco-BRL), 0.5 mM ascorbic acid (Sigma), and 0.45 mM monothioglycerol (Sigma). On the second day of differentiation, the EBs were treated with 50 ng/mL activin A to stimulate endoderm differentiation. On day 4, the EBs were treated with 50 ng/mL BMP-4, 10 ng/mL basic FGF, and 50 ng/mL activin A. On day 7, the EBs were collected, rinsed with PBS, and seeded on gelatin-coated plates in a specialized EB medium. This medium was formulated without ascorbic acid and monothioglycerol but supplemented with 10 ng/mL EGF (Peprotech), 10 ng/mL basic FGF (Peprotech), 20 ng/mL HGF (Peprotech), 20 ng/mL TGF-β1 (R&D Systems), 10 ng/mL VEGF (R&D Systems), and 100 nM dexamethasone (Sigma). The EBs were cultured for 10 or 14 days.

### Maintenance and differentiation of human iPSCs

A human iPSC line 201B7 was obtained from RIKEN BioResource Research Center Cell Bank and maintained on MMC-treated MEFs in a medium of DMEM/Ham’s F12 supplemented with 20% KSR, 2 mM l-glutamine, 0.1 mM NEAA, 1 × P/S, 0.1 mM 2-mercaptoethanol, and 5 ng/ml human basic FGF. In feeder-less cultures, iPSCs were cultured on SyntheMax II (Corning)-coated dishes with StemFit medium (AK02N, Ajinomoto) to maintain the primed state. One day before and after passages, 10 μM Y27632 (BLD pharm) was added to the cells to mitigate cell death.

For the random differentiation, human iPSCs cultured in feeder-less conditions were dissociated into single cells with Accutase (Nacalai Tesque), and seeded at a density of 6 × 10^3^ cells/well on Nunclon Sphera 96-well U-bottom plates using DMEM/Ham’s F12-based iPSC medium without basic FGF and with 10 μM Y27632. After one day, half of the medium was replaced with fresh iPSC medium without basic FGF and Y27632. The EBs were maintained for 3 or 6 days, with the medium being replaced every 2 days.

The differentiation of iPSCs into endodermal epithelial cells and hepatocytes was done using an established protocol.^16^ Briefly, human iPSCs were dissociated into single cells using accutase and seeded at a density of 6 × 10^4^ cells/mL on Nunclon Sphera 96-well U-bottom plates using RPMI1640 supplemented with 1 × N2 and B27 to promote EB formation. The differentiation process involved several stages. On day 1, iPSCs were treated with 10 ng/mL activin A and 12 ng/mL FGF4 for 2 days, along with 0.1% heat-inactivated FBS. From days 3-5, the cells were treated with a mixture of 100 ng/mL activin A, 20 ng/mL FGF4, and 10 ng/mL BMP4 in a 2% heat-inactivated FBS environment. On day 6, EBs were transferred to a Matrigel-coated dish and cultured with advanced DMEM/F12, supplemented by 1 × N2, 1 × B27, and 2 mM l-glutamine. This was followed by a 3-day treatment with 50 ng/mL FGF10. Starting from day 9, the cells were subjected to 50 ng/mL FGF10, 0.1 μM retinoic acid, and 10 μM SB431542 for an additional 2 days. From day 11 onward, a final phase of treatment involved 30 ng/mL FGF10, 50 ng/mL EGF, and 50 ng/mL HGF, continuing until day 20. Immunofluorescent staining and qRT-PCR were performed on days 5, 8, 10, and 20. Primed iPSCs cultivated on SyntheMax II-coated plates were used as baseline samples for day 0. The assessments included immunofluorescence staining of frozen EB sections from days 5, 8, and 10.

### Establishment of an *Oct4* promoter-*egfp* mouse ESC line.

To generate an *Oct4* promoter-*egfp* (*Oct4*-*egfp*) reporter plasmid, the mouse proximal promoter region of *Oct4* gene (approximately 2.0 kb) was amplified by PCR using genome DNA extracted from mouse ESC line D3 (Supplementary Table S1). The PCR reaction was performed with LA-Taq (Takara). The amplified fragment was subcloned into AseI and NheI sites of a promoter-less enhanced green fluorescent protein expressing vector, pEGFP-c1 (Clontech). Mouse ESCs (D3) were transfected with 5 μg of linearized plasmid by lipofectamine 2000 (Invitrogen). Two days later, 500 μg/mL G418 (Sigma) was added to the culture medium to select the ESC lines stably expressing EGFP protein. Well-separated ESC colonies were picked up and maintained in the above mouse ESC maintenance medium supplemented with 500 μg/mL G418 and used for the following analyses.

### Knock-down of *Epha2* and overexpression of human *EPHA2* in mouse ESCs

Short hairpin double-strand DNA (Supplementary Table S1) were annealed and subcloned into BamHI and ClaI sites of a retrovirus vector pSINsi-DK I (TAKARA). For the overexpression of *EPHA2*, human *EPHA2* cDNA was amplified by PCR and inserted into XhoI and NotI sites of pMYs-IRES-puro retroviral vector (Cell Biolabs). The vectors were propagated in DH5*α* and purified by Midiprep kit (MACHEREY-NAGEL) according to the manufacturer’s protocol. Retroviral packaging Plat-E cells were transfected with the plasmids using PEI-MAX (Polysciences). The retroviruses secreted to the culture medium were precipitated with PEG8000 and incubated with dissociated mouse ESCs (D3) in an ESC maintenance medium in the presence of 8 μg/mL polybrene. The cells were infected with the virus by centrifugation at 500 × *g* for 30 minutes at 35 °C and seeded onto a gelatin-coated dish. The stably infected cells were selected with 500 μg/mL G418 for pSINsi-DK I vector-infected *Epha2* knocked-down cells and with 0.5 μg/mL puromycin for pMYs vector-infected human *EPHA2* overexpressing cells, respectively. The RNA interference and exogenous expression were confirmed by qRT-PCR.

### Preparation of RNA and protein samples

Total RNAs were extracted with ISOGEN (Nippon Gene) according to the manufacturer’s instructions. Single-strand cDNA was synthesized from 500 ng of the total RNA using PrimeScript 1st Strand cDNA Synthesis kit (Takara) according to the manufacturer’s instruction. Quantitative reverse-transfer polymerase chain reaction (qRT-PCR) was performed in 10 μL of mixture with 1 μL of each cDNA, Taq polymerase, dNTP, SYBR Green PCR Master Mix, and the respective gene-specific primer sets (Supplementary Table S1). PCR was performed for 40 cycles of denaturation at 95 °C for 15 seconds and annealing/extension at 60 °C for 30 seconds.

The protein extract was prepared by homogenization in a protein extraction buffer that contained 50 mM Tris–HCl (pH7.4), 150 mM NaCl, 1.0% (w/v) NP40, and protease inhibitor cocktail (Complete, Roche). After centrifugation at 27 000 × *g* for 5 minutes at 4 °C, the supernatants were collected as the whole cell extracts. The protein concentrations were measured with a protein assay kit (Bio-Rad Laboratories).

### Alkaline phosphatase staining

Mouse ESCs were fixed with 4% PFA in PBS for 5 min at room temperature, washed with PBS, and incubated with an alkaline phosphatase (AP) substrate, BM purple AP substrate (Roche), for 30 minutes at room temperature to visualize the enzyme activity.

### Antibodies

For immunofluorescent staining, the following primary antibodies were used: rabbit polyclonal anti-mouse and human OCT4 (sc-9081; Santa Cruz Biotechnology), mouse monoclonal anti-OCT4 (sc-5279; Santa Cruz Biotechnology), rabbit polyclonal anti-mouse NANOG (RCAB0001P; REPROCELL), mouse monoclonal anti-mouse SSEA-1 (TM13; Kyowa Medex), rabbit polyclonal anti-mouse EPHA2 (sc-924; Santa Cruz Biotechnology), mouse monoclonal anti-β-TUBULIN (T5293, Sigma), goat polyclonal anti-mouse AFP (sc-8108; Santa Cruz Biotechnology), and goat anti-mouse and human ALB (55727; MP Biomedicals). For FACS analysis of mouse ESCs, the following primary antibodies were used: monoclonal anti-mouse CRIPTO antibody (MAB1538; R&D systems), anti-mouse SSEA1-APC (FAB2155A; R&D systems), anti-mouse TRA-1-60 (ab16288; Abcam), and monoclonal anti-mouse EPHA2 conjugated to phycoerythrin (PE) (FAB639P; R&D systems). For immunofluorescent analyses of human iPSCs, mouse monoclonal anti-human EPHA2 (MAB3035; R&D systems) and goat polyclonal anti-human SOX17 (AF1924, R&D systems) were used. Regarding the secondary antibodies for immunofluorescent staining, Alexa Fluor 488 donkey anti-mouse IgG (H + L) (A21202; Invitrogen), Alexa Fluor anti-goat IgG (H + L) (A11055; Invitrogen), and Alexa Fluor 594 donkey anti-rat IgG (H + L) (A21209; Invitrogen) were used. For MACS, monoclonal Rat antiMouse EPHA2 (LSC36250; LSBio) was used as the 1st antibody, and Dynabeads-conjugated sheep anti-Rat IgG (Invitrogen) was used as the 2nd antibody.

### Flow cytometry analysis

Mouse cells were dissociated with 0.5 mM EDTA and 1 mg/mL of collagenase in either PBS supplemented with 20% KSR (Gibco) and 1 mM CaCl_2_ or Gibco Cell Dissociation Buffer enzyme-free Hanks’-balanced salt solution for 5 min. The cells were then Fc-blocked with anti-rat CD32 (BD Pharmingen), resuspended in ice-cold 2% FCS/PBS, and incubated with the following 1st antibodies, anti-EPHA2 (1/100), anti-EPHA2-PE (1/400), anti-TRA-1-60 (1/200), anti-CRIPTO (1/100), or anti-SSEA1-APC, (1/200) dilution, for 1 hour at room temperature. After washing, the cells were further incubated with 2nd antibody (1/100 dilution) for 1 hour at room temperature. FACS sorting was done with FACSAria Cell Sorter (BD Falcon) and analyzed with FACSDiva software (BD Falcon) and FlowJo software (Treestar). Human 201B7 cells were dissociated with 0.5 mM EDTA, blocked with 2% donkey serum in ice-cold PBS, and incubated with human EPHA2 antibody at 1/100 concentration for 1 hour at room temperature without fixation and permeabilization. Dead cells were visualized by 7-ADD staining. Using a cell sorter MA900 (SONY), the cells were directly sorted into a tube supplemented with 1 mL of ISOGEN for RNA extraction and qRT-PCR analysis. For the culture of EPHA2^+^ and EPHA2^-^ cells, the sorted cells were harvested in iPSC maintenance medium with 5 ng/mL basic FGF, 10 μM Y27632, and 1 × P/S, and cultured on MMC-treated MEFs.

### Magnetic cell sorting and in vivo transplantation

The hepatocytes differentiated from WT and *Oct4* promoter-*egfp* mouse D3 ESCs were dissociated with trypsin/EDTA for 5 minutes. The cells were resuspended in 2% FCS/PBS and incubated with EPHA2 antibody-conjugated magnetic beads for 1 hour at room temperature. The cells (1.0 × 10^6^) were fractionated with MACS and analyzed by FACS to monitor the efficiency of MACS-based fractionation. As a control, the differentiated cells derived from *Oct4-egfp* reporter ESCs were plated on gelatin-coated dish and cultured for 7 days in ESC maintenance medium to monitor the contamination of undifferentiated cells after differentiation.

For in vivo transplantation, MACS-sorted 1.0 × 10^6^ cells differentiated from D3 ESCs (days 10 or 14) were injected into the testicular subcutaneous tissue or portal veins of 6-week-old male SCID mice (CB17/IcrJcl-Prkdc^scid^ mice, purchased from CLEA Japan). Differentiated hepatocytes incubated without 1st antibody but with magnetic beads were also sorted with MACS as the negative control. At 3 months after the injection, the transplanted cells grown in the SCID mice were harvested and paraffin-embedded for histological and immunofluorescent staining.

### In silico study of single-cell RNA sequencing and statistical analyses

A publicly accessible single-cell RNA sequencing dataset of mouse early embryonic development (GEO accession: GSE121650),^17^ mouse iPSC reprogramming (GSE137050),^18^ and undifferentiated human ESC H1 and H9 data subset (GSE75748)^19^ was analyzed by an R package “Seurat.”^20^ The single cells were clustered based on the expression of human *EPHA2* expression levels. For the statistical analyses, the unpaired *t*-test or chi-square test was used to evaluate statistical differences between 2 groups. The analysis of variance followed by Tukey–Kramer or Dunnett’s tests was performed by an R package “multcomp,”^21^ when 3 or more groups were compared. Means were considered significantly different when *P* value was <.05.

### Ethics

All the DNA recombination experiments were approval by the DNA recombination experiment committees of AIST and NAIST. All animal experiments were approval by the animal experiment committee of AIST and performed following the institutional animal experiment guidelines.

## Results

### Undifferentiated state-specific expression of EPHA2 on the cell surface of mouse ESCs

**Figure 1.**
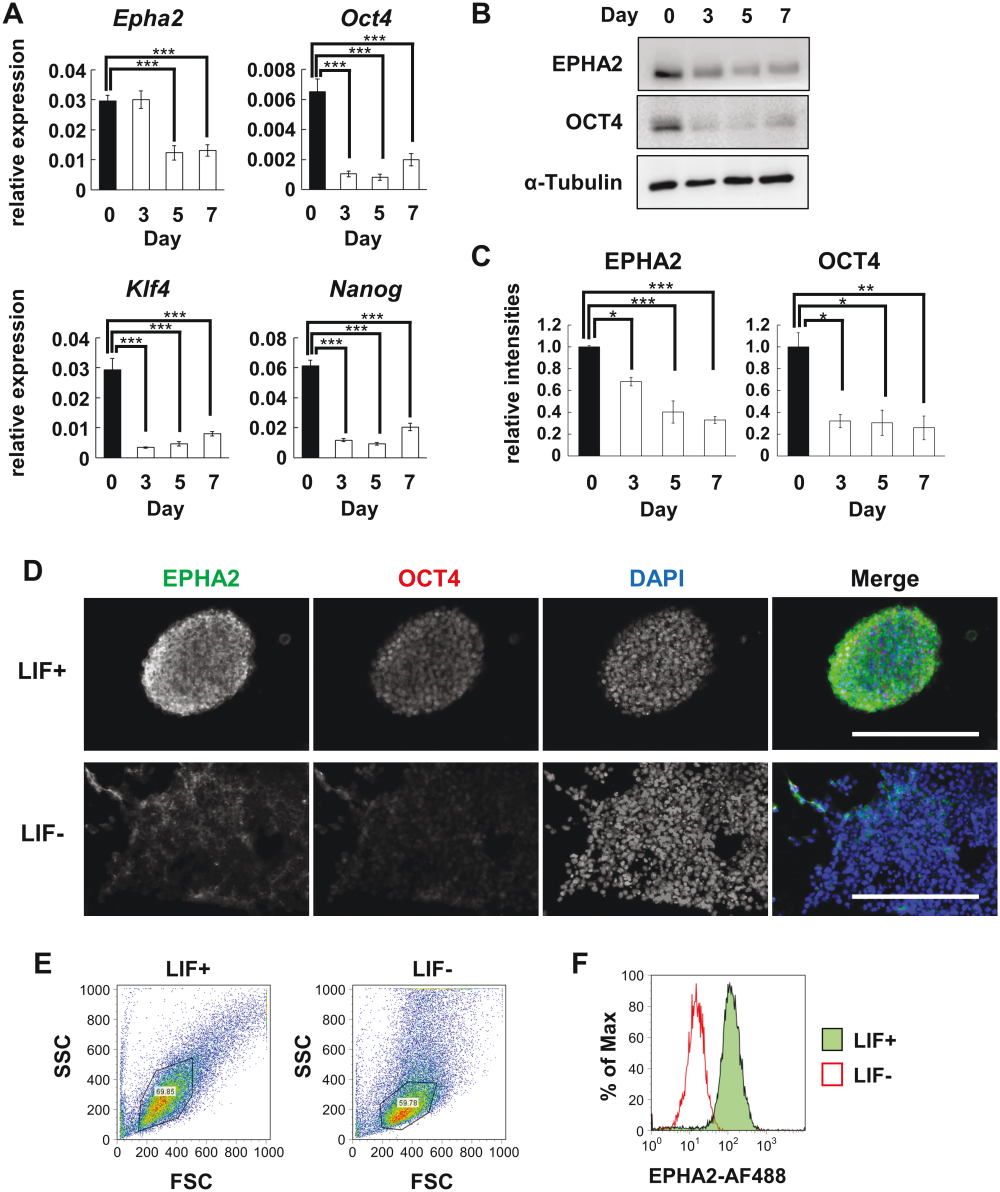
EPHA2 is expressed on the cell surface of undifferentiated mouse ESCs and downregulated during differentiation. (A) Relative gene expression of *Epha2* mRNA in mouse ESCs (D3) cultured without LIF for 0, 3, 5, and 7 days. (B) Immunoblotting of EPHA2 protein of mouse ESCs cultured as in (A). (C) EPHA2 protein levels in (B) normalized to α-Tubulin. (D) Immunofluorescent staining of mouse ESCs. The cells were cultured with or without LIF for 7 days. Bars; 200 μm. (E, F) Flow cytometric analysis of EPHA2 protein on the cell surface of mouse ESCs. Living cells were stained with an EPHA2 antibody. The propidium iodide^+^ dead cells were removed from the analysis. FSC and SSC profiles (E) and the histogram of EPHA2-AF488 levels (F) of the gated cells in (E) were shown. Statistical analyses in (A) and (C) were calculated by Dunnett’s test. Data were graphed as means ± SE of 3 independent biological replicates. *P < .05; **P < .01; ***P < .001.

As we previously reported,^12^ EPHA2 expression diminishes following the removal of LIF, evidenced at both transcript (Figure 1A) and protein levels (Figure 1B and 1C). NCBI database epigenetic data confirms active transcription of promoter regions of *Epha2* gene in pluripotent mouse ESCs, with binding of undifferentiated state-specific transcription factors like OCT4 (Supplementary Figure 1A). A correlation emerges between the expressions of *Epha2* and *Oct4* in mouse ESCs (Figure 1A-1C), substantiating the relationship between these 2 elements. Prior scRNA-seq analyses of early embryos have shown that *Epha2* is highly expressed at the blastocyst stage, with its expression diminishing as development progresses (Supplementary Figure 1B).^17^ Similarly, an increase in *Epha2* expression following the reprogramming of mouse iPSCs has been observed in additional scRNA-seq studies (Supplementary Figure 1C).^18^ This suggests that *Epha2* serve as a marker of pluripotent stem cells in mice. Immunofluorescent staining (Figure 1D) coupled with flow cytometric analyses (Figure 1E and 1F) corroborates the presence of EPHA2 on the cell surfaces of undifferentiated mouse ESCs, offering additional layers of verification.

### *Epha2* knock-down induced spontaneous differentiation of mouse ESCs

**Figure 2.**
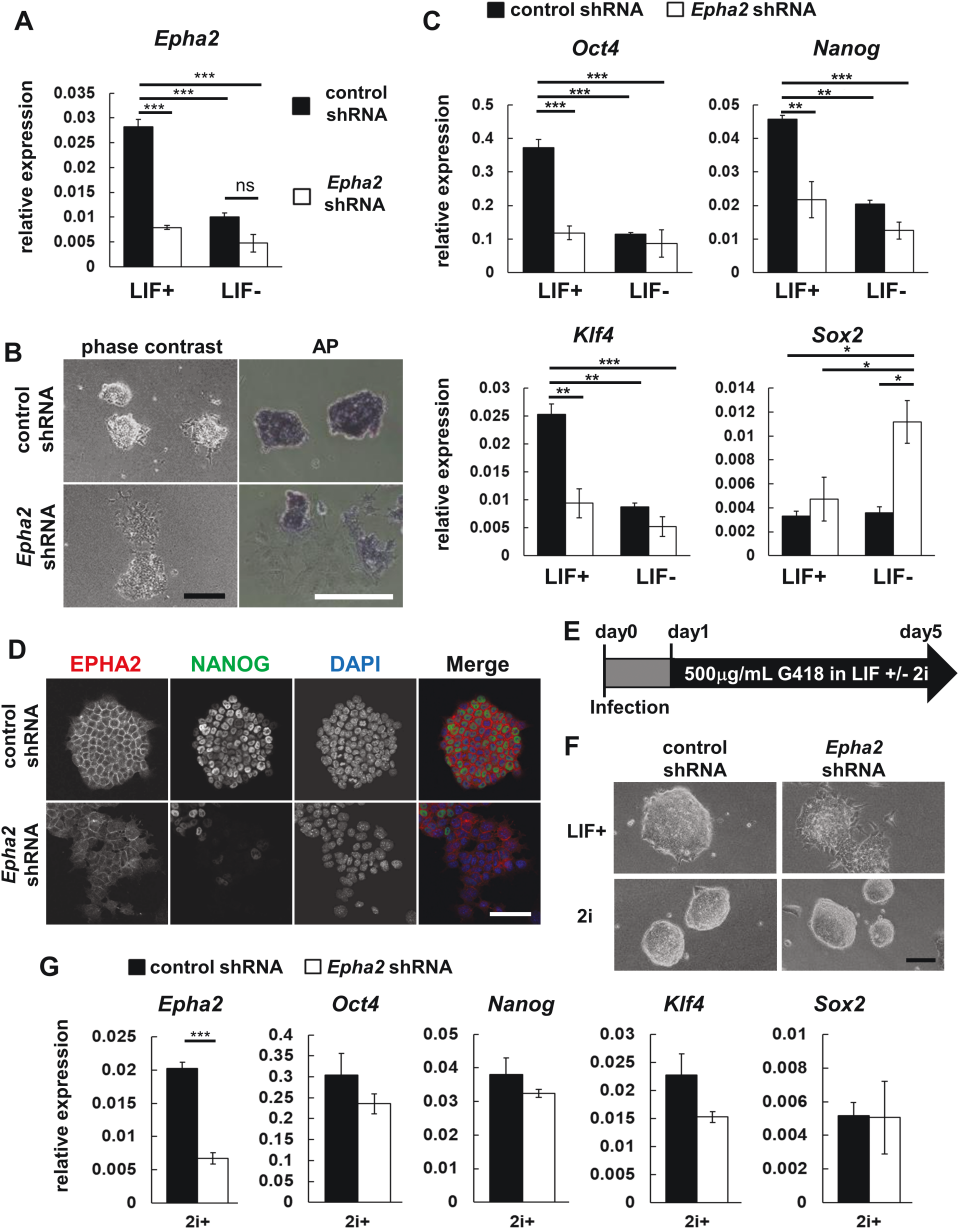
Knock-down of *Epha2* induces spontaneous differentiation of mouse ESCs. (A) qRT-PCR analysis of *Epha2* mRNA after knock-down in mouse ESCs (D3). The cells were infected with *Epha2* shRNA retrovirus and cultured with or without LIF for 7 days. (B) Images of phase contrast and alkaline phosphatase (AP) staining of *Epha2* KD mouse ESCs cultured in ES maintenance medium with LIF for 5 days. Bars; 200 μm. (C) qRT-PCR analysis of undifferentiated state-specific marker genes in *Epha2* KD mouse ESCs. (D) Immunofluorescent staining of *Epha2* KD mouse ESCs cultured with LIF for 7 days. Bars; 100 μm. (E) Culture conditions of *Epha2* KD mouse ESCs after infection with *Epha2* shRNA retrovirus. One day after infection with *Epha2* shRNA virus, mouse ESCs were cultured with or without 2i in the presence of G418 and LIF for 4 days. (F) Phase contrast images of *Epha2* KD mouse ESCs cultured as in (E). Bar; 200 μm. (G) qRT-PCR analysis of *Epha2* and undifferentiated state-specific marker genes in *Epha2* KD mouse ESCs cultured with 2i. All qRT-PCR analyses were performed with 3 independent biological replicates and graphed as means ± SE. The significant differences were calculated by Tukey test in (A and C) and *t*-test in (G). *P < .05; **P < .01, ***P < .001.

We proceeded to explore the role of EPHA2 in mouse ESC pluripotency and differentiation. Establishing *Epha2* knock-down (KD) ESC lines via RNA interference (Figure 2A) revealed notable changes. In the KD clones, the round-shaped mouse ES colonies on a gelatin-coated dish nearly vanished, and undifferentiated state-specific AP activity diminished even in the presence of LIF (Figure 2B). Undifferentiated state-specific markers were also suppressed in KD cells (Figure 2C and 2D), indicating loss of *Epha2* triggers spontaneous differentiation, unaffected by LIF. Subsequent analysis demonstrated that spontaneously differentiated *Epha2*-KD cells expressed marker genes representative of each of the 3 germ layers (Supplementary Figure S2), suggesting that EPHA2 does not selectively inhibit differentiation in a particular lineage. The overexpression of human *EPHA2* in *Epha2*-KD mouse ESCs restored cell morphology (Supplementary Figure 3A and 3B) and endogenous *Oct4* expression levels (Supplementary Figure 3C and 3D). Interestingly, 2i medium-containing MAPK inhibitor PD0325901 and GSK3β inhibitor CHIR99021–prevented *Epha2* KD-induced differentiation (Figure 2E-2G). These findings imply the role of EPHA2 in inhibiting MAPK or GSK3 signaling, independent of LIF. Further examination of the role of EPHA2 in differentiation revealed that when *Epha2*-KD ESCs underwent differentiation into 3 germ layers via EB formation (Supplementary Figure 4A and 4B), there was a heightened induction of differentiation markers in KD cells (Supplementary Figure 4C-4E). This underscores the pivotal role of EPHA2 as a regulator that not only preserves their undifferentiated state but also inhibits ESC differentiation.

### EPHA2^+^ subpopulation of human undifferentiated iPSCs exhibited transient state with high OCT4 and NANOG expression

**Figure 3.**
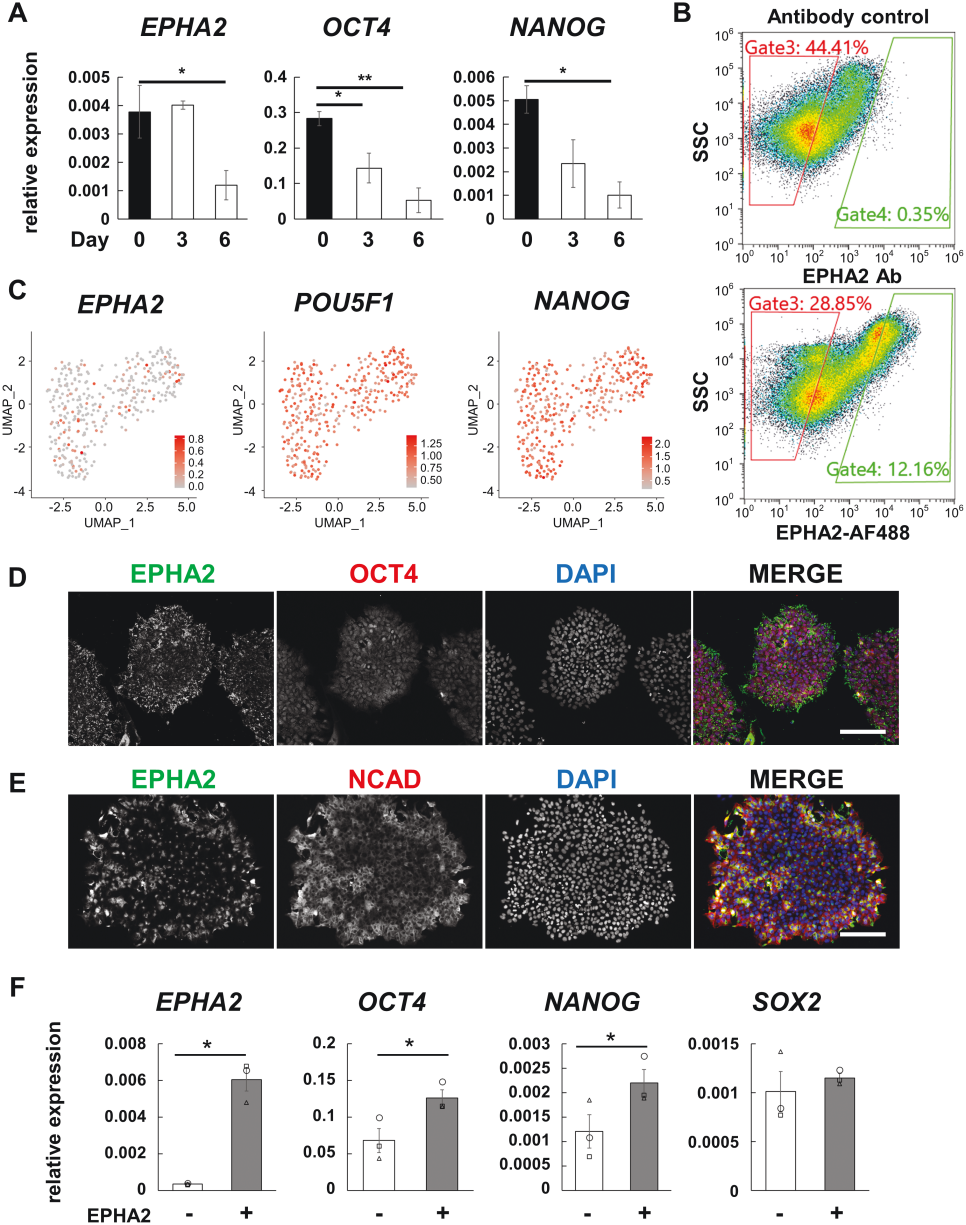
Expression of EPHA2 in heterogenous subpopulation of human PSCs. (A) qRT-PCR analysis of *EPHA2* and undifferentiated state-specific marker genes during EB-based random differentiation of human iPSCs (201B7) without basic FGF. Statistical analysis was done by Dunnett’s test comparing to day 0 and graphed as means ± SE of 3 independent experiments. *P < .05; **P < .01. (B) Representative flow cytometric plots of living human iPSCs stained with EPHA2 antibody-conjugated with AFF488. Gate3 and Gate4 were sorted as EPHA2^−^ and EPHA2^+^ cell populations, respectively. See also Supplementary Figure 10A. (C) Feature plots of *EPHA2* and undifferentiated state-specific genes in publicly available undifferentiated human ESC H1 and H9 data subsets from GSE75748 scRNA-seq dataset. Note that *EPHA2* expression in hESCs was heterogeneous. Normalized expression levels were plotted. (D, E) Immunofluorescent staining of human iPSC cultured on SyntheMax II-coated plate with StemFit medium. The cells were fixed with paraformaldehyde in PBS and permeabilized. Bars; 200 μm. (F) qRT-PCR analysis of fractioned EPHA2^+^ and EPHA2^-^ subpopulations. EPHA2^+^ cells express higher *OCT4* and *NANOG*, than EPHA2^−^ cells. Means ± SE of 3 independent experiments were shown. Statistical significance was defined as *P < .05 by *t*-test.

We also examined the expression pattern of human EPHA2 under the undifferentiated state. *EPHA2* mRNA levels markedly declined following the spontaneous differentiation of human iPSCs, mirroring the trend observed in mouse ESCs (Figure 3A). However, FACS analysis revealed that approximately 12% of undifferentiated iPSCs displayed heterogeneous EPHA2 expression on the cell surface (Figure 3B). This undifferentiated state-specific heterogeneous expression pattern was also observed in human ESCs, as evidenced by analysis of public single-cell RNA sequencing datasets of human ESC H1 and H9 lines^19^ (Figure 3C), indicating the presence of a distinct EPHA2^+^ subpopulation within human PSCs. Immunofluorescent staining indicated prominent EPHA2 detection around the periphery of paraformaldehyde-fixed and permeabilized iPSC colonies cultured in feeder-less conditions (Figure 3D), similar to the distribution of the previously identified founder cell marker, N-cadherin.^22^ However, the distribution of N-cadherin did not entirely coincide with EPHA2 (Figure 3E).

Analysis of genomic and epigenetic data revealed a high degree of conservation in the EPHA2 loci of both human and mouse, including adjacent gene orders (Supplementary Figures 1 and 5). In the human EPHA2 locus, active enhancer regions in iPSCs^23^ harbor multiple binding sites for OCT4 and NANOG (Supplementary Figure 5). Corroborating this, EPHA2^+^ PSCs exhibited heightened *OCT4* and *NANOG* expression, but not *SOX2*, compared to EPHA2^-^ cells (Figure 3F). Morphological distinctions between EPHA2^+^ and EPHA2^−^ cells were inconspicuous under both on-feeder and feeder-less culture conditions (Supplementary Figure 6A). Notably, post-passage EPHA2^+^ cells swiftly regenerated EPHA2^-^ cell populations (P2 in Supplementary Figure 6B and C), and the elevated expression of *OCT4* dissipated (Supplementary Figure 6D). Collectively, these findings suggest that EPHA2-high cells are emblematic of a transient state characterized by augmented *OCT4* and *NANOG* expression.

### EPHA2 identifies ESC-derived teratoma-forming cells

Next, we aimed to explore the concurrent expression of EPHA2 and OCT4 in undifferentiated cells. A mouse ESC line, marked with *Oct4* promoter-*egfp* (*Oct4*-*egfp*), was established to visualize *Oct4* expression in the undifferentiated state. This marker, EGFP, was strongly expressed in undifferentiated cells but its expression diminished when the cells were differentiated by the withdrawal of LIF (see Supplemental Figure 7A and 7B).

Then, we employed EPHA2 antibody-based MACS to separate EPHA2^+^ cells from a mixture of *Oct4-egfp* ESCs, which were cultured either with or without LIF over a span of 7 days. This process resulted in a significant reduction of EGFP^+^ cells (Supplementary Figure 7C). From these observations, we deduce that EPHA2 serves as a potent cell surface marker for identifying undifferentiated mouse ESCs.

**Figure 4.**
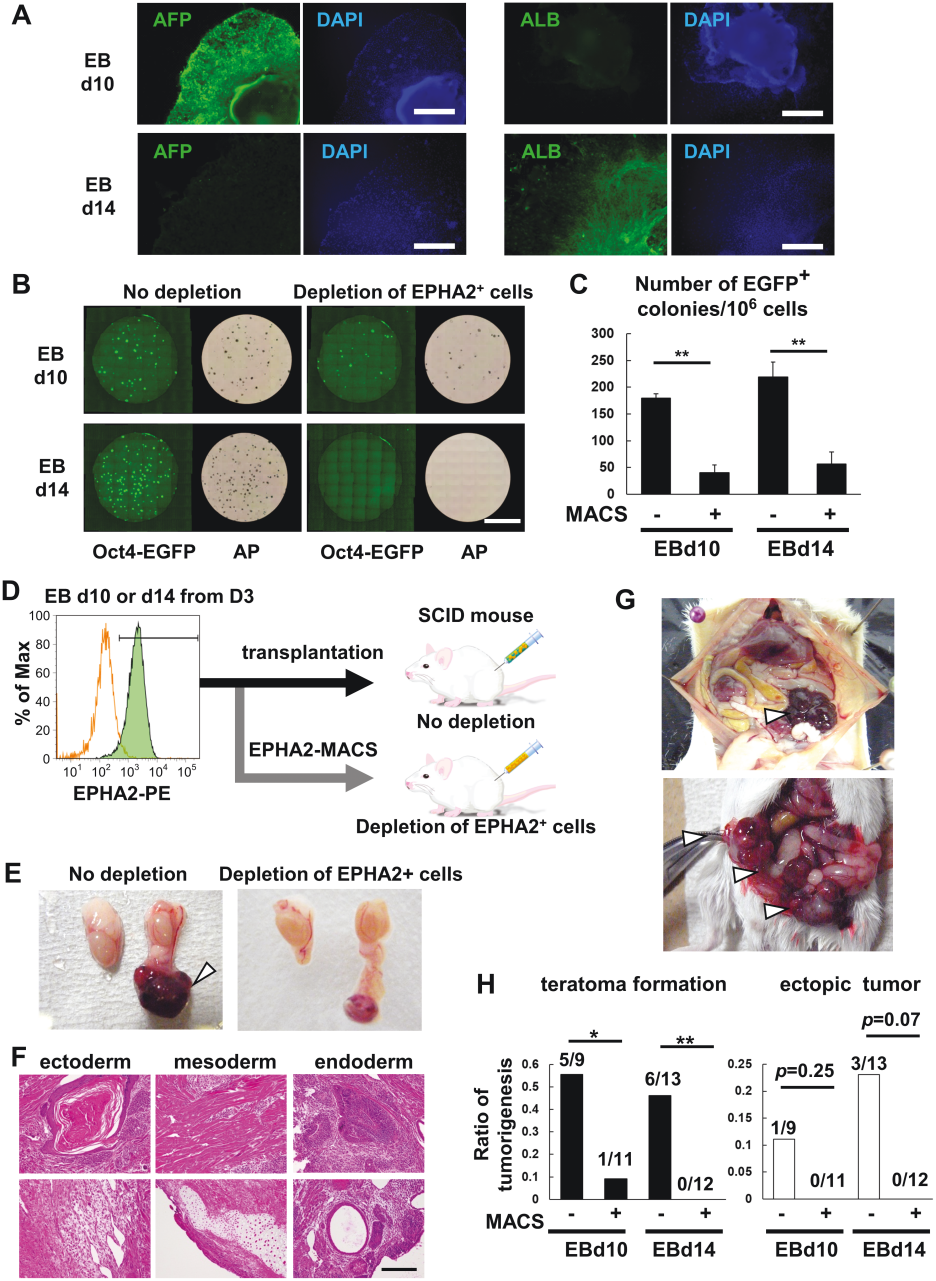
Transplantation of EPHA2^+^ cells into immune-deficient mice formed tumors in vivo. (A) Immunofluorescent staining of mouse EBs differentiated into hepatocyte lineages. Expression of an early hepatocyte marker AFP at day 10 and a mature marker ALB at day 14 were detected. Bars; 200 μm. (B) Depletion of undifferentiated ES colonies after removal of EPHA2^+^ cells from EBs. *Oct4-egfp* ESCs were differentiated by EB formation for 10 and 14 days. EPHA2^+^ cells were removed from EBs using anti-EPHA2 antibody-bound MACS after dissociation with trypsin/EDTA. The residual cells were cultured in ES maintenance medium with LIF for 7 days. The alkaline phosphatase (AP) activity was visualized by incubating with AP substrate. Bar; 2 cm. (C) The number of EGFP^+^ cell colonies in (B). Statistical analysis was done by Tukey test and graphed as means ± SE of 4 independent experiments. (D) Scheme of in vivo transplantation experiment. Mouse ESCs (D3) were differentiated into hepatocyte linages and the EBs were dissociated by EDTA treatment. The cells were transplanted into SCID mice after depletion of EPHA2^+^ cells by MACS. (E) Decreased teratoma formation after transplantation of EPHA2^−^ cells. White arrowheads indicate teratomas. (F) H&E staining of teratomas formed in the testicular subcutaneous tissue without MACS procedure. Typical cell types of 3 germ layers were confirmed. Bar; 500 μm. (G) Typical teratoma formation after transplantation of dissociated EB at day 10 through hepatic portal vein. White arrowheads indicate teratomas. (H) Quantification of teratoma formation in (E) and (G). Statistical analysis was done by Chi-square test, *P < .05, **P < .01.

We investigated whether EPHA2 serves as a marker for undifferentiated cells after EB-based differentiation of ESCs. Utilizing a well-established differentiation protocol,^24,25^ we induced mouse ESCs into the hepatocyte lineage (Supplementary Figure 8A). By days 10 and 14, the induction of hepatocyte precursor marker alpha fetoprotein^14^ and mature hepatocyte marker Albumin^25^ were observed respectively (Figure 4A). We noted that approximately 1% of EGFP^+^ cells remained at day 14 when *Oct4-egfp* ESCs underwent the same differentiation process (Supplemental Figure 8B and 8C). This observation indicates the persistence of undifferentiated cells even after extended differentiation. When these differentiated EBs were trypsinized into single cells and cultured in ESC maintenance medium, EGFP^+^ colonies emerged within a week. Interestingly, the depletion of EPHA2^+^ cells using anti-EPHA2 antibody-bound MACS led to a decrease in these Oct4-EGFP^+^ colonies (Figure 4B and 4C).

Considering the tumorigenic risks of undifferentiated cells when transplanted into immune-deficient mice, we differentiated the ESC D3 line into hepatocytes, processed the cells with and without EPHA2-MACS depletion, and introduced them into SCID mice (Figure 4D). After 4 weeks of post-testicular subcutaneous transplantation, teratoma formation, consisting of 3 germ layer lineages, was evident (Figure 4E and 4F). Teratoma formation was also observed in the vesicular glands around the intestine following transplantation through the hepatic portal vein (Figure 4G). This confirmed the presence of undifferentiated cell contamination even after prolonged induction of differentiation. However, the removal of EPHA2^+^ cells by EPHA2-MACS significantly mitigated teratoma formation (Figure 4H), indicating EPHA2 as a viable antigen for identifying teratoma-forming cells within differentiated cell populations.

Additionally, we observed no impact on the engraftment and maturation of transplanted cells in vivo, which were ALB^+^ following the removal of EPHA2^+^ cells (Supplementary Figure 8D). Our findings were corroborated upon confirming EPHA2 expression in other mouse ESC lines, E14 and B6G2. Similar to the D3 cell line, EPHA2 expression diminished during hepatocyte differentiation (Supplementary Figure 8E and 8F). This evidence underscores the potential of EPHA2 as a novel cell surface antigen for tumorigenic cells in mice.

### EPHA2 is a potential cell surface marker in OCT4^+^ human iPSCs

**Figure 5.**
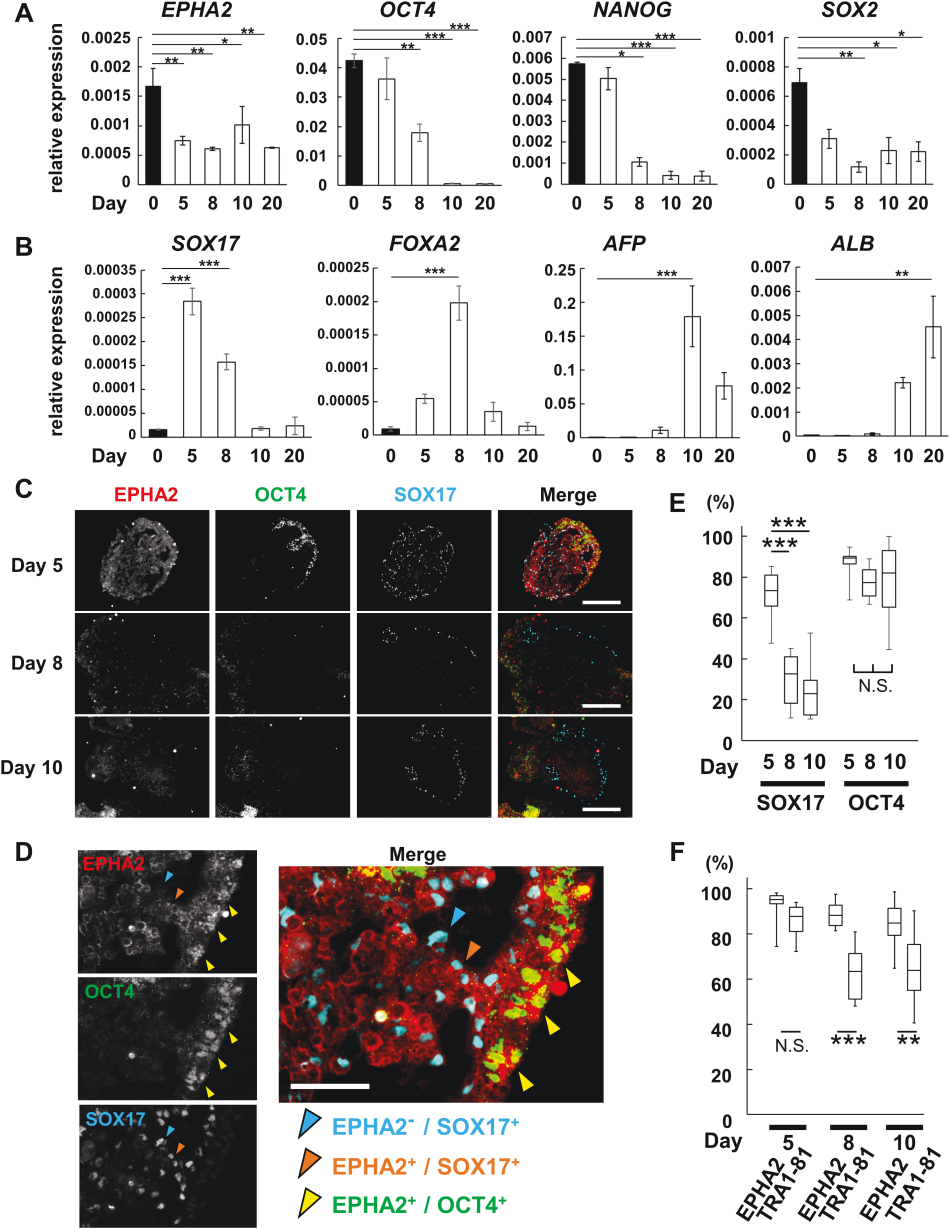
Co-expression of EPHA2 with OCT4 in EBs during human iPSC differentiation into hepatocyte. (A, B) Relative gene expression of undifferentiation and differentiation markers during hepatic induction. Statistical analysis was done by Dunnett’s test against day 0 and graphed as means ± SE of 3 independent biological replicates. *P < .05; **P < .01, ***P < .001. (C) Immunofluorescent staining of EBs at days 5, 8, and 10. Bars; 200 μm. (D) Enlarged images of EB at day 5 in (C). Bars; 50 μm. (E, F) Quantification of immune-positive cells in Supplementary Figure 9E. Box plot showing the percentage of EPHA2^+^ cells among SOX17^+^ or OCT4^+^ cells (E). Box plot showing the percentage of EPHA2^+^ and TRA1-81^+^ cells among OCT4^+^ cells (F). Each box represents 1st quartile, median, and 3rd quartile, and whiskers show the minimum and maximum values. Ten images of independent EBs were analyzed. Total count of DAPI^+^ nuclei per image were between 1 × 10^3^ and 2 × 10^3^. Statistical significance was defined by Tukey test of SOX17 and OCT4, respectively in (E) and *t*-test between EPHA2 and TRA1-81 in (F). **P*p* < .01, ***P < .001, N.S; no significance between 3 with *P* > .05.

EPHA2 expression was also noted in OCT4^+^ human iPSCs. During hepatocyte differentiation of human iPSC 201B7 using the EB-based method (Supplementary Figure 9A and 9B), *EPHA2* and undifferentiated state-specific markers like *OCT4*, *NANOG*, and *SOX2* showed reduced expression (Figure 5A). In contrast, early endoderm markers *SOX17* and *FOXA2* transiently increased, followed by hepatic markers *AFP* and *ALB* (Figure 5B and Supplemental Figure 9C and 9D). EPHA2^+^/OCT4^+^ cells were identified within EBs at day 5 (Figure 5C), primarily not co-expressing early endoderm marker SOX17 (Figure 5C and 5D). Although not every EPHA2^+^ cell was OCT4^+^ in later stages, the majority of OCT4^+^ cells did express EPHA2 during differentiation (Figure 5E). The detection of Oct4^+^ undifferentiated cells by EPHA2 was comparable to that by TRA1-81 at day 5 but superior to that by TRA1-81 at days 8 and 10 (Figure 5F). EPHA2^+^ cells, isolated by flow cytometry (Supplementary Figure 10A and 10B), maintained elevated *OCT4* and *NANOG* mRNA levels, marking undifferentiated cells during hepatocyte lineage differentiation (Supplementary Figure 10C-10E). Consequently, the strong correlation between EPHA2 and OCT4/NANOG expressions in both mouse and human PSCs, even post-differentiation, positions EPHA2 as a crucial pluripotency regulator and a prospective cell surface antigen for undifferentiated and tumorigenic stem cells.

## Discussion

While pluripotent stem cells hold promise for regenerative medicine, concerns persist about tumorigenic risks due to PSC contamination.^26,27^ It is generally recognized that many tumors resulting from the transplantation of undifferentiated cells are benign teratomas. However, even mouse ESCs with a normal karyotype possess the potential to develop teratocarcinomas upon transplantation into immunodeficient mice.^28^ Because of remarkable similarities between these PSCs and tumor cells, such as rapid proliferation rate, lack of contact inhibition, high telomerase activity, as well as high expression of oncogenes such as MYC and KLF4, there exists a risk scenario wherein the presence of undifferentiated PSC contaminating the transplantation process into human recipients may lead to the development of malignant teratocarcinomas, rather than benign teratomas.^29^ Thus, for effective stem cell therapy, it is essential to eliminate tumorigenic cells from differentiated cells before transplantation.^30,31^

In our research, we observed pronounced expression of EPHA2 on the surface of various mouse ESC lines. We also identified human EPHA2 in undifferentiated OCT4^+^ cells within human iPSC-derived hepatocytes. Utilizing established in vitro methods to differentiate both mouse and human PSCs into hepatocytes,^14-16^ we effectively removed undifferentiated cells capable of forming teratomas from these differentiated populations using an EPHA2-specific antibody. Given our findings, we suggest EPHA2 as a robust surface marker for identifying residual undifferentiated cells, potentially reducing tumorigenic risks post stem cell transplantation.

*LIN28A* is one of the most sensitively silenced genes in differentiated cells, allowing for the detection of residual undifferentiated cells using droplet digital PCR in human iPSC-derived cardiomyocytes.^32^ Additionally, significant upregulation of mouse *Lin28a* has been observed during the induction of iPSCs, comparable to *Epha2* (Supplementary Figure 1C). However, LIN28A is an intracellular protein, posing challenges in removing undifferentiated cells for transplantation. Traditionally, carbohydrate antigens such as SSEAs and TRA1-60/81, along with the recently identified R-10G and R-17F,^33,34^ have been employed as cell surface markers in detecting pluripotent stem cells. Since these TRA1-60/81, R-10G, and R-17F glycan epitopes are all present on a common core protein, Podocalyxin (PODXL),^33,34^ we compared EPHA2 with one of these markers, TRA1-81. However, TRA1-81 was less efficient than EPHA2 in identifying residual undifferentiated OCT4^+^ cells within human iPSC-derived hepatocytes, especially in the later stages of differentiation (Figure 5F), highlighting the need for more effective cell surface markers. Thus, we propose the combined use of EPHA2 and these traditional markers, which could lead to even more extensive removal of residual undifferentiated cells for transplantation.

Ephrin receptors are notable in both tumor progression^35^ and embryonic development.^36^ As EPHA2 is recognized to be expressed and functional in certain tissues such as blood vessels,^37^ notochord,^38^ and skin,^39^ as evidenced by mouse experiments, its applicability to these tissues is subject to limitations. While EPHA2 exhibits modest expression in other normal human tissues, it is prevalent in tumor tissues^40,41^ and cancer cell lines.^42^ Moreover, elevated EPHA2 levels are linked with poor prognosis in various cancers.^40^ This suggests a role of EPHA2 in tumor cell migration, invasion, and metastasis.^40,43^ Given that EPHA2 is classified as a type I transmembrane protein with a substantial extracellular domain, it stands as a promising drug target.^35^ However, the precise mechanisms linking EPHA2 to tumor cells and PSCs remain to be fully elucidated.

In this research, we revealed the role of EPHA2 in preserving mouse ESCs pluripotency. Our findings also show that the absence of *Epha2* in mouse ESCs greatly promotes differentiated marker expression, indicating the role of *Epha2* in safeguarding stem cells against differentiation. Spontaneous differentiation observed after *Epha2*-KD (Supplementary Figure 2) and the differentiation of *Epha2*-KD cells via EB formation (Supplementary Figure 4) consistently resulted in the prominent induction of the early ectoderm marker *Sox1*, mesendoderm marker *T*, early mesoderm marker *Mixl1*, and early endoderm marker *Gata6*. These findings suggest that EPHA2 does not selectively suppress differentiation toward a specific lineage, but instead impedes differentiation at a stage preceding germ layer specification. Previous study on *Epha2* knock-out mouse ESCs^44^ proposed minimal impact on mouse ESC pluripotency. Conversely, our findings highlighted spontaneous differentiation in mouse ESCs post *Epha2* knock-down. This difference might stem from the compensatory *Epha1* elevation in the *Epha2* knock-out ESCs from the earlier study, possibly overshadowing the phenotype.^45^ However, similar to our results, their *Epha2* KO ESCs did show accelerated differentiation marker induction including *T*.^44^ Thus, the potential function of EPHA2 in PSCs might be to limit ESC differentiation.

We propose that EPHA2^+^ cells represent a distinct subset of human PSCs marked by elevated *OCT4* and *NANOG* expression. It is known that primate PSCs exhibit transcriptional heterogeneity, a phenomenon also noted in post-implantation embryos.^46,47^ While the combined functions of OCT4, NANOG, and SOX2 are established in mouse ESCs,^48^ these transcription factors appear to drive specific lineage commitments differently in primate PSCs.^49^ A recent study highlighted pluripotent founder cells that express N-Cadherin at the fringes of primate PSC colonies in vitro, drawing parallels to the in vivo traits of primitive endoderm in primate epiblast.^22^ Our immunofluorescence assays using EPHA2 and N-Cadherin antibodies on human iPSCs did not show complete overlap, suggesting that EPHA2^+^ cells differ from the primitive endoderm. It is possible that the EPHA2^+^ subset reflects a specific transitional state of PSCs around the time of human embryo implantation.

In this study, we revealed that both mouse and human PSCs with EPHA2 expression have heightened levels of OCT4 and NANOG. Furthermore, EPHA2 can act as a marker for tumorigenic undifferentiated cells during PSC differentiation. This discovery has potential implications for mitigating the tumorigenic risks associated with PSCs in regenerative medicine.

## Conclusions

This research positions EPHA2 as a promising cell surface marker for undifferentiated and tumorigenic stem cells in both mouse and human. The potential of EPHA2 to mitigate the risk of tumorigenesis highlights the significance in advancing the safety of stem cell transplantation for regenerative medicine applications.

## Supplementary material

Supplementary material is available at *Stem Cells Translational Medicine* online.

szae036_suppl_Supplementary_Figures_S1-S10

szae036_suppl_Supplementary_Table_1

## Data Availability

The plasmids used in this study, data supporting the findings, and the R codes for the computational analyses of this study can be obtained from the corresponding author upon reasonable request.
