# Correction to: EPHA2 is a novel cell surface marker of OCT4-positive undifferentiated cells during the differentiation of mouse and human pluripotent stem cells

**DOI:** 10.1093/stcltm/szae054

**Published:** 2024-07-11

This is a correction to: Atsushi Intoh *et al.*, EPHA2 is a novel cell surface marker of OCT4-positive undifferentiated cells during the differentiation of mouse and human pluripotent stem cells, *Stem Cells Translational Medicine*, 2024; szae036, https://doi.org/10.1093/stcltm/szae036

Acknowledgment of funding support from the Koyanagi-Foundation was omitted from the originally published version and has been added.

