# Supplementary material for: EPHA2 is a novel cell surface marker of OCT4-positive undifferentiated cells during the differentiation of mouse and human pluripotent stem cells
Source: Stem Cells Transl Med. 2024 May 29;13(8):763–75. doi: 10.1093/stcltm/szae036 (PMC11328934; doi:10.1093/stcltm/szae036)
Supplement: szae036_suppl_Supplementary_Figures_S1-S10 [file szae036_suppl_supplementary_figures_s1-s10.zip › Supplementary Figures Intoh 240426/Supplementary Figures Intoh 240426.docx]

# Supplemental information

## Supplemental Figure 1: Genomic and epigenetic information of mouse *Epha2* gene in Ensembl database.

(A) *EphA2* locus in mouse chromosome 4. Note that the promoter regions of this gene are active in mouse ESC lines (filled box). Typical undifferentiated state-specific transcription factor (TF) binding sites are shown. Transcription start site (TSS). (B, C) Violin plots of *Epha2* and undifferentiated state-specific genes in publicly available scRNA-seq dataset. (B) Mouse embryonic development (GSE121650) from embryonic day E4.5-5.5 to E7.5. (C) Mouse iPSC reprogramming (GSE137050) from MEF, day 5, day 10, and established iPSCs. Normalized expression levels were plotted.

## Supplemental Figure 2: qRT-PCR analysis of differentiation marker genes in EphA2-KD ESCs.

(A-C) Induction of differentiation marker genes for ectoderm (A), mesoderm (B), and endoderm (C) in *Epha2*-KD mouse ESCs cultured with LIF for 7 days were measured by qRT-PCR analysis. All qRT-PCR analyses were performed by three independent experiments, and the statistical analysis was performed by *t*-test between control and KD groups. ND: not detected; *; *p* < 0.05; **; *p* < 0.01; ***; *p* < 0.001.

## Supplemental Figure 3: Rescue of *Epha2* knock-down by overexpression of human *EPHA2* in mouse ESCs.

(A) Culture conditions of mouse ESCs infected with retroviruses expressing mouse *EphA2* shRNA and human *EPHA2* cDNA. One day after infection with these retroviruses, the ESCs were selected with puromycin and G418 in the presence of LIF. (B) Phase contrast images of mouse ESCs infected with the indicated retroviruses. No obvious morphological difference was observed between the control and human *EPHA2*-overexpresing (OE) cells but not mouse *Epha2* knockdown (KD) cells. Bars; 200 μm. (C) Typical gene expressions in (B). The gene expressions were analyzed by qRT-PCR. Means ± SE of three independent experiments. *; p < 0.05. (D) Immunofluorescent staining of mouse ESCs in (B). Recovery of OCT4 was observed by the overexpression of human *EPHA2*. Bars; 100 μm.

## Supplemental Figure 4: Knockdown of *Epha2* promoted the differentiation of mouse ESCs.

(A) EB-based differentiation into three germ layers of mouse *Epha2*-KD ESCs. The ESCs maintained in the presence of LIF and 2i were differentiated as indicated. The phase contrast pictures of ESC colonies before differentiation were shown below. Bars; 200 μm. (B) qRT-PCR analysis of undifferentiated state-specific marker genes at day 7 after differentiation. (C-E) Induction of differentiation marker genes for ectoderm (C), mesoderm (D), and endoderm (E) in the differentiated *Epha2* KD cells. All qRT-PCR analyses were performed by three independent experiments, and the statistical analysis was performed by *t*-test between control and KD groups in (B-E). *; *p* < 0.05.

## Supplemental Figure 5: Genomic and epigenetic information of human *EPHA2* gene in Ensembl database.

Human *EPHA2* gene is located on chromosome 1, and 20kb upstream and 15kb downstream of the gene were shown. Note that the promoter regions of this gene were active in human ESCs (pink box), and typical undifferentiated state-specific transcription factor (TF) binding sites were found in these regions. One silencer region was found^23^, where FOXA2 binding site were identified (blue box).

## Supplemental Figure 6: Transient state of human EPHA2-high PSCs with enhanced expression of *OCT4* and *NANOG*

(A) Phase contrast images of the FACS sorted EPHA2^+^ and EPHA2^-^ cells (P1). No significant morphological change was observed between EPHA2^+^ and EPHA2^-^ fractions. Bars; 200 μm. (B) Flow cytometric analysis of human iPSCs (P0) and passaged EPHA2^-^ and EPHA2^+^ populations (P1 and P2). Plots of EPHA2 and the histograms were shown on the right side. (C) Percentages of EPHA2 high cells gated in (B) were graphed as means ± SE of three independent experiments. Different letters indicate statistically significant differences with *p* < 0.05 by Tukey test. (D) qRT-PCR analysis of undifferentiated state-specific genes in P1 and P2 populations. The control cells were plated without sorting. Means ± SE of three independent experiments were shown, Tukey test within each passage number were calculated. *; *p* < 0.05; **; *p* < 0.01.

## Supplemental Figure 7: Depletion of EPHA2^+^ cells by MACS using an *Oct4* promoter-*egfp* mouse ESC line.

(A) Cell morphology and EGFP expression of an *Oct4* promoter-*egfp* (*Oct4-egfp*) mouse ESC line cultured with or without LIF for 7 days. Bar; 100 μm. (B) Flow cytometric analysis of *Oct4-egfp* mouse ESCs cultured with or without LIF for 7 days. (C) Depletion of EPHA2^+^ cells by MACS using anti-EPHA2 antibody. *Oct4-egfp* ESCs cultured with or without LIF for 7 days were combined and EPHA2^+^ cells were removed by MACS. The resulted fractions were analyzed by flow cytometry. Depletion of EPHA2^+^ cells by MACS showed significant decrease of Oct4-EGFP^+^ cells.

## Supplemental Figure 8: Expression of EPHA2 during the differentiation of mouse ESCs into hepatocytes.

(A) Culture conditions for the differentiation of mouse ESCs into hepatocyte lineage. (B, C) Residual undifferentiated Oct4-EGFP^+^ cells even after differentiation. *Oct4-egfp* reporter mouse ESCs were differentiated into hepatocyte and the dissociated cells were analyzed by flow cytometry at day 10 and day 14. EGFP^+^ cells were detected in EBs even at day 14. See also Figure 4A-C. (D) Immunofluorescent staining of ALB in engrafted cells derived from day 14 differentiation of mouse D3 ESCs. Bars; 200 μm. (E, F) Flow cytometric analysis of the other ESC lines E14 and B6G2 after differentiation. The expression of EPHA2, SSEA1, and CRIPTO in (E) E14 and (F) B6G2 cell lines were analyzed before and after hepatic differentiation.

## Supplemental Figure 9: Expression of EPHA2 during hepatocyte differentiation of human iPSCs.

(A) Schematic overview of EB-based hepatocyte differentiation of human iPSCs. Definitive endoderm was induced by an EB-based method in a U-bottom low cell attachment plate (upper scheme) from day 0 to day 5. EBs were further cultured on Matrigel-coated dish and differentiated into hepatocyte lineage (lower scheme). (B) Phase contrast images of iPSC-derived EBs under suspension culture and 2D culture on Matrigel-coated dish. Bars; 1 mm. (C) qRT-PCR analysis of marker genes during hepatocyte differentiation. The statistical analysis was done by Dunnett’s test against day 0 and graphed as means ± SE of three independent experiments. *; *p* < 0.05; **; *p* < 0.01, ***; *p* < 0.001. (D) Immunofluorescent staining of EBs cultured on Matrigel-coated dish at day 20. Weak signal of EPHA2 was detected in the outgrowth area. Bars; 200 μm. (E) Immunofluorescent staining of human iPSC-derived EBs with OCT4 and SOX17 antibodies together with EPHA2 or TRA1-81 antibodies. The consecutive sections at day 5, 8, and 10 were compared and graphed in Figure 5E and F. Bar; 200 μm.

## Supplemental Figure 10: Co-expression of *EPHA2* with *OCT4* and *NANOG*.

(A) Flow cytometric analysis of EPHA2 in living human iPSCs during differentiation. EPHA2^-^ (Gate3, red) and EPHA2^+^ (Gate4, green) cells in 7-ADD-unstained viable population (Gate2) were analyzed at day 5, 8, and 10. The histograms representing the upper FACS plots were delineated with different colors: gray for the negative control, green for day 0, and blue for days 5, 8, and 10. (B) qRT-PCR analysis of *EPHA2* in EPHA2 high and low cells at day 5, 8, and 10. (C-E) qRT-PCR analysis of undifferentiation and differentiation-specific genes. Gene expressions were normalized by *GAPDH*. The expression of *NANOG* at day 10 was not detected. Samples were collected from three independent experiments and graphed as means ± SE. Statistical significance was calculated by *t*-test. *; *p* < 0.05; **; *p* < 0.01.
