# Supplementary material for: EPHA2 is a novel cell surface marker of OCT4-positive undifferentiated cells during the differentiation of mouse and human pluripotent stem cells
Source: Stem Cells Transl Med. 2024 May 29;13(8):763–75. doi: 10.1093/stcltm/szae036 (PMC11328934; doi:10.1093/stcltm/szae036)

Supplemental Figure 1

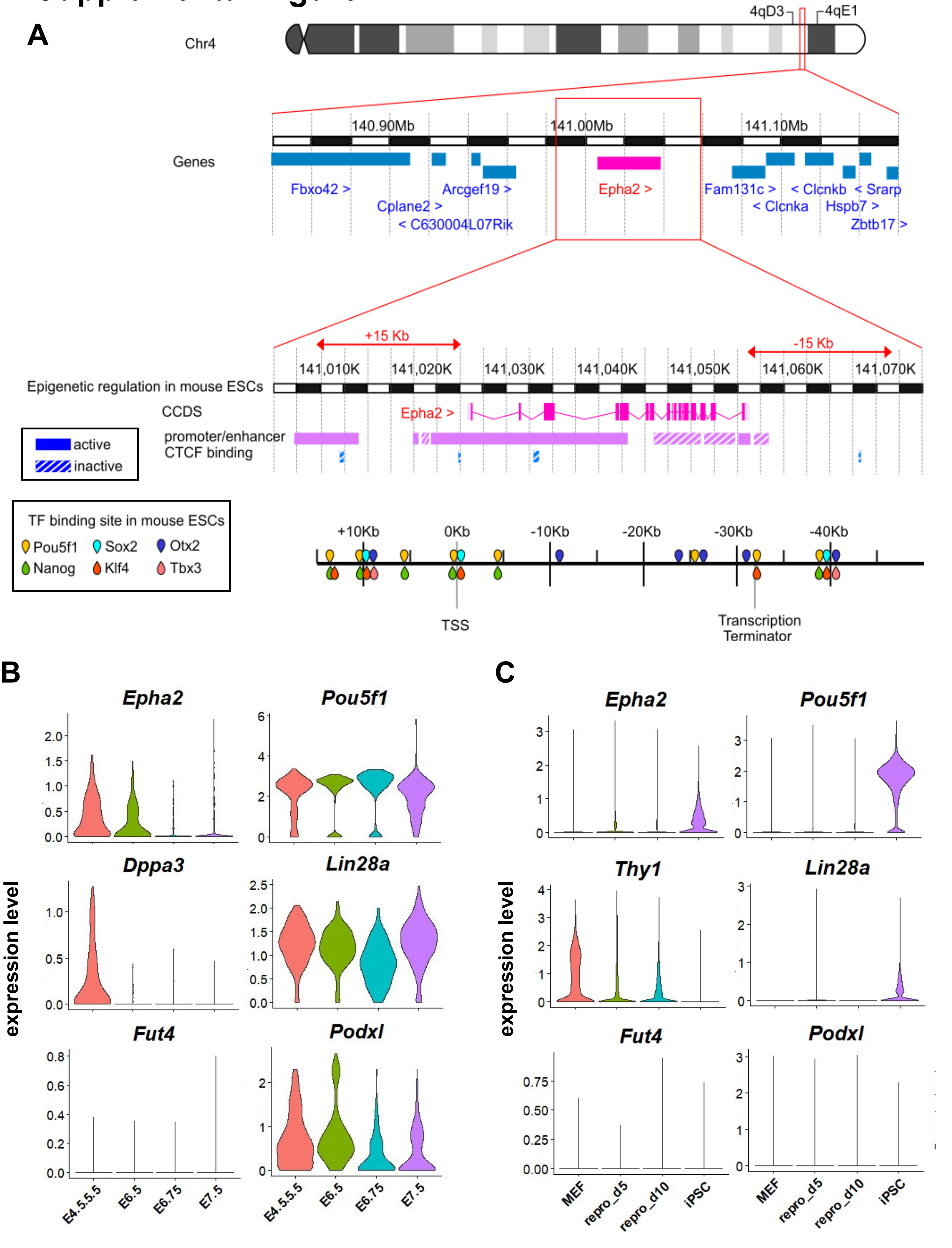

Supplemental Figure 2

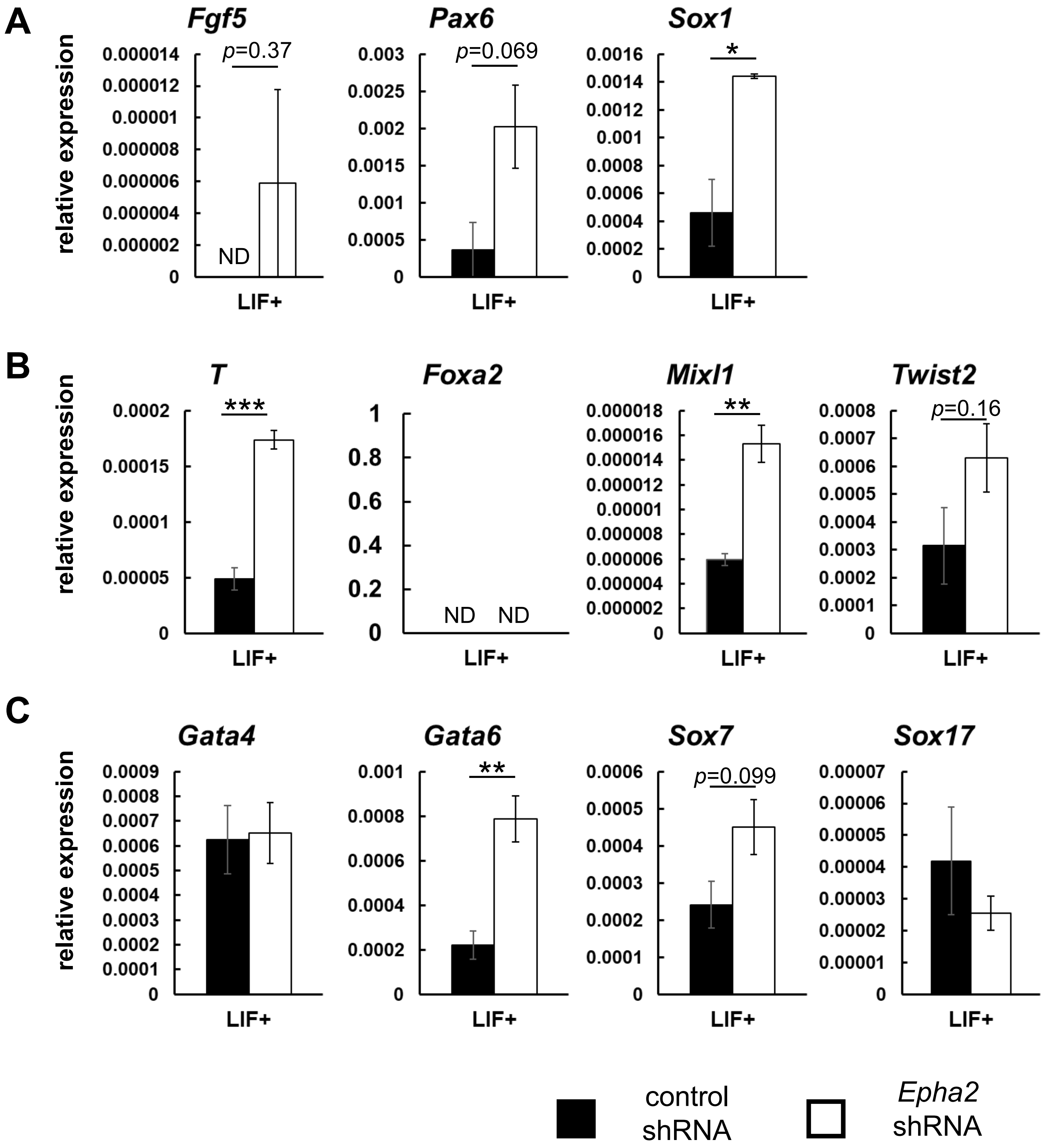

Supplemental Figure 3

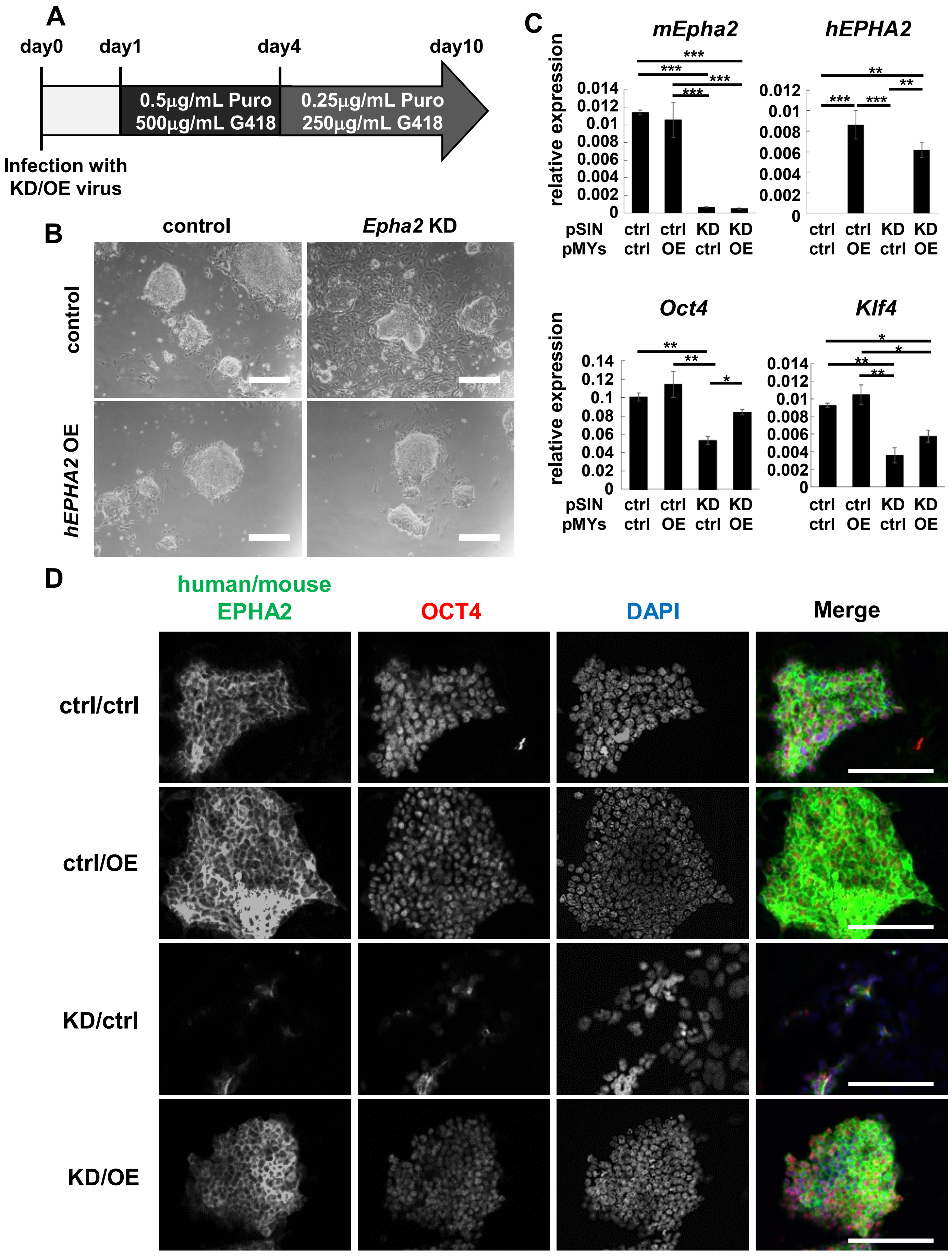

Supplemental Figure 4

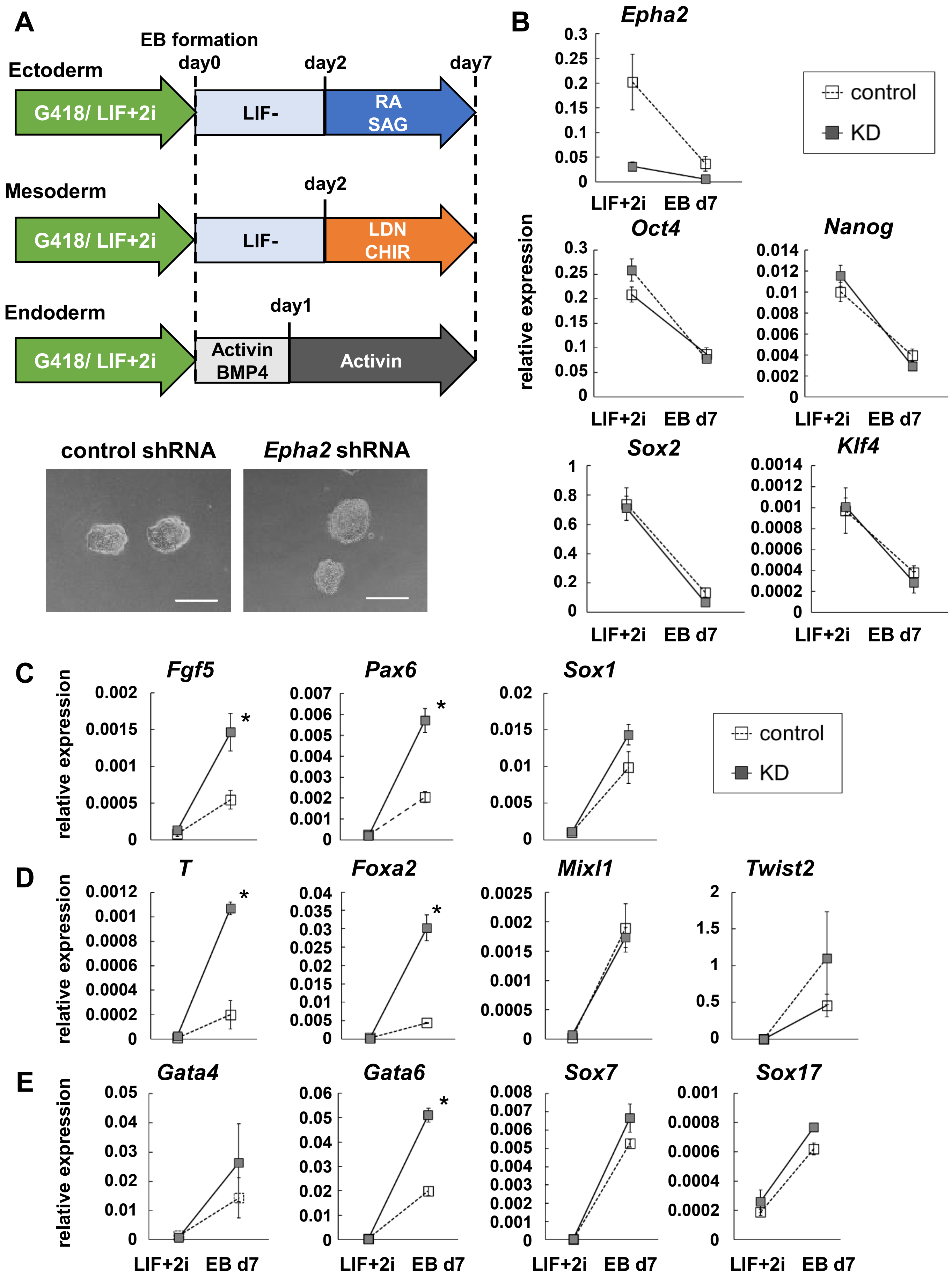

Supplemental Figure 5

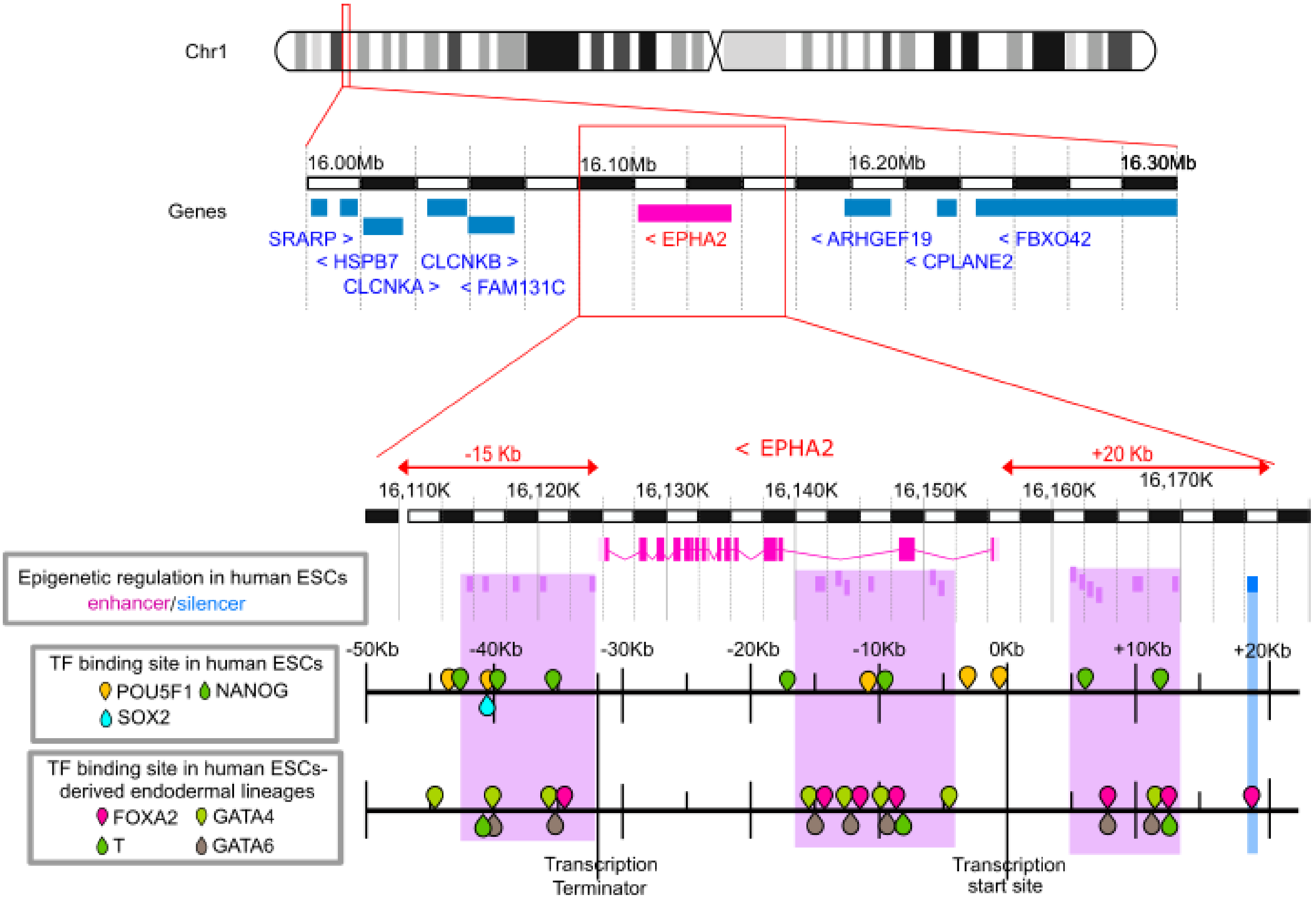

Supplemental Figure 6

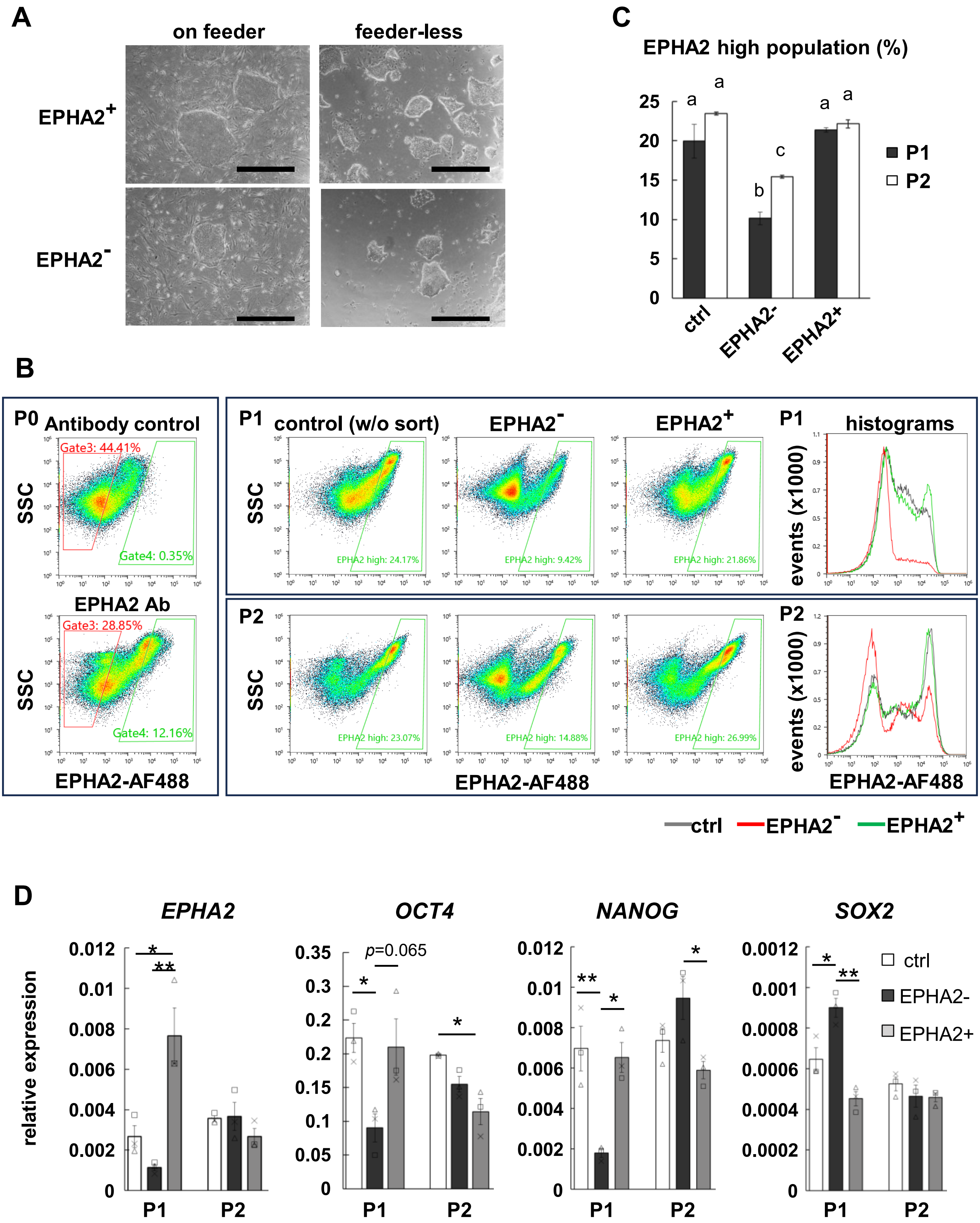

Supplemental Figure 7

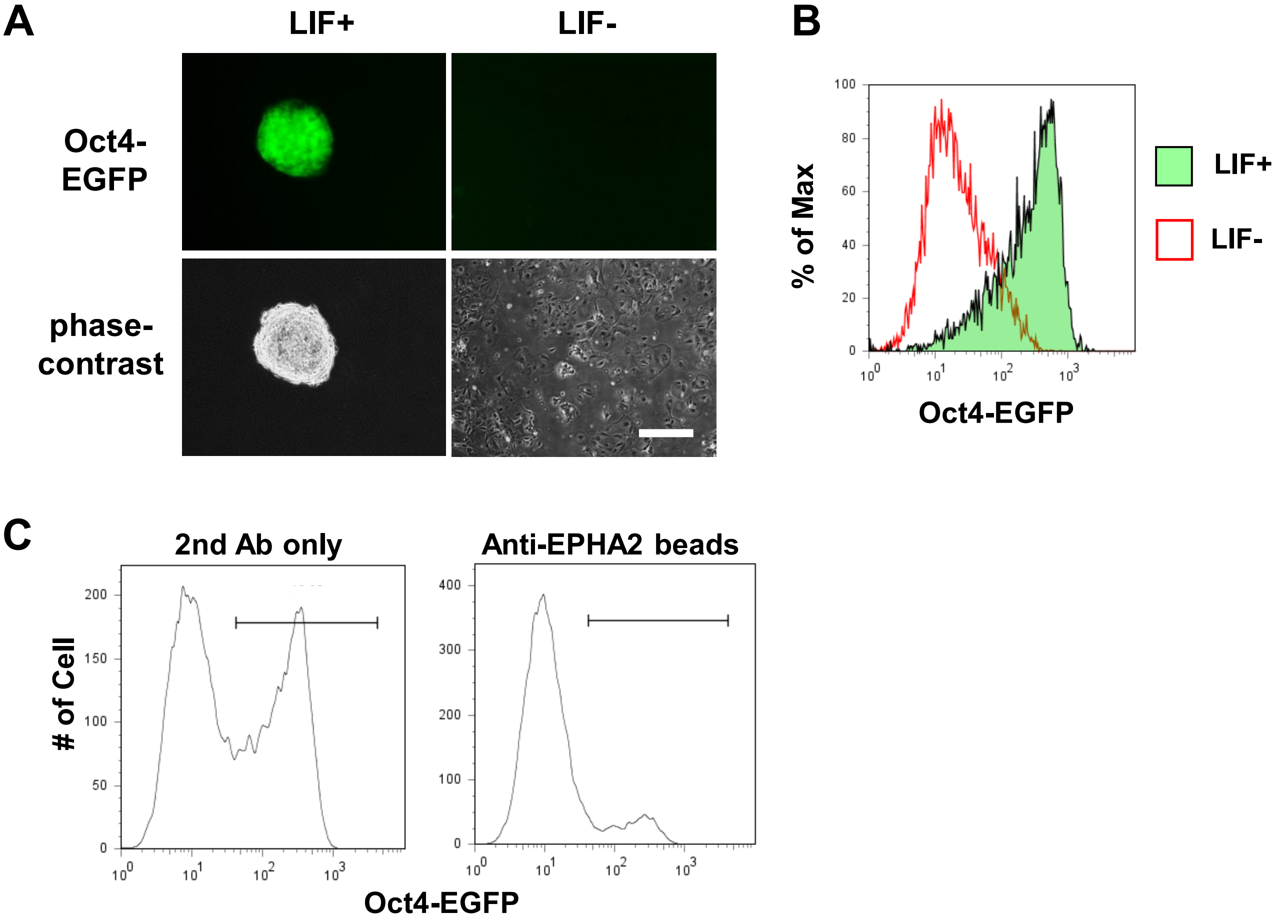

Supplemental Figure 8

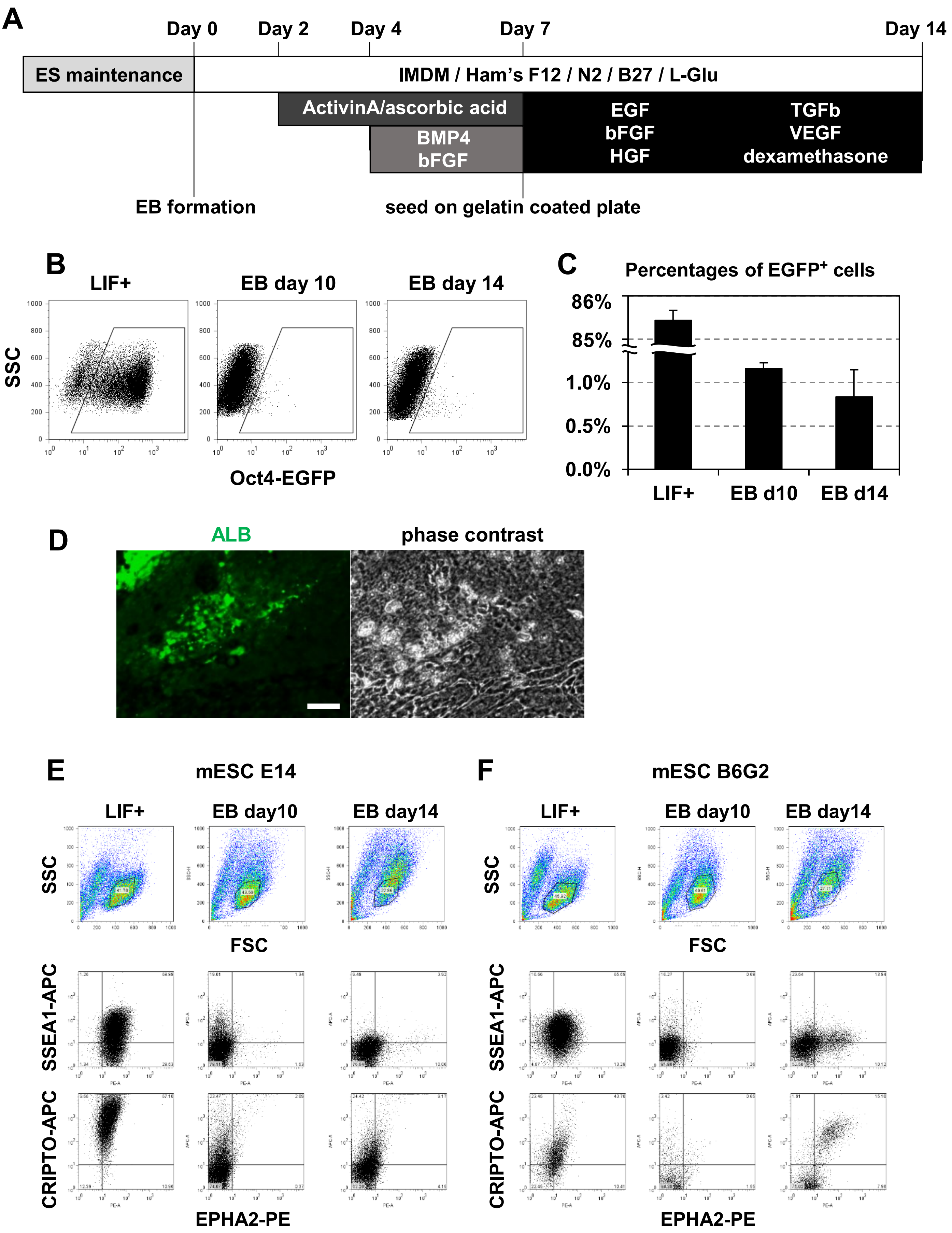

Supplemental Figure 9

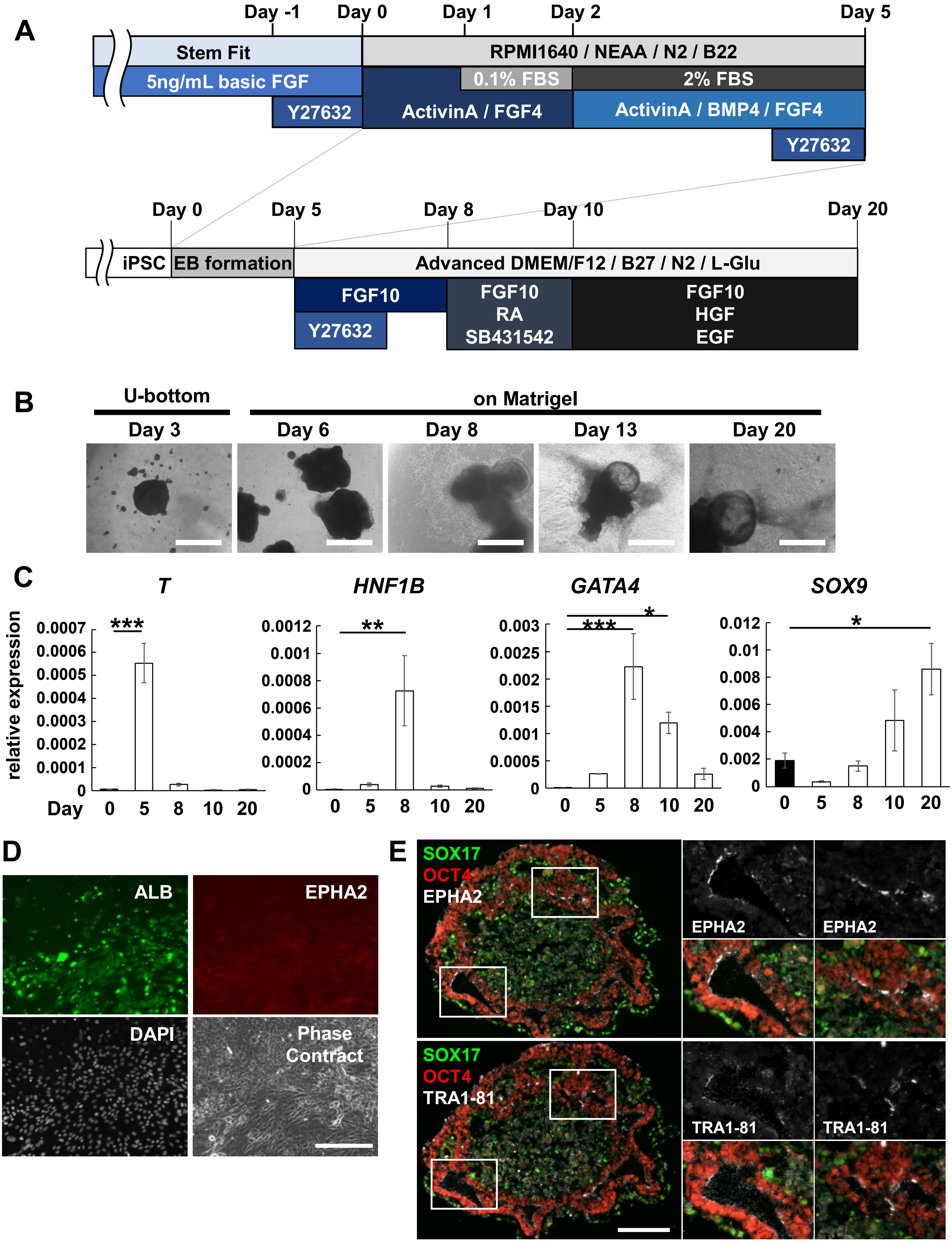

Supplemental Figure 10

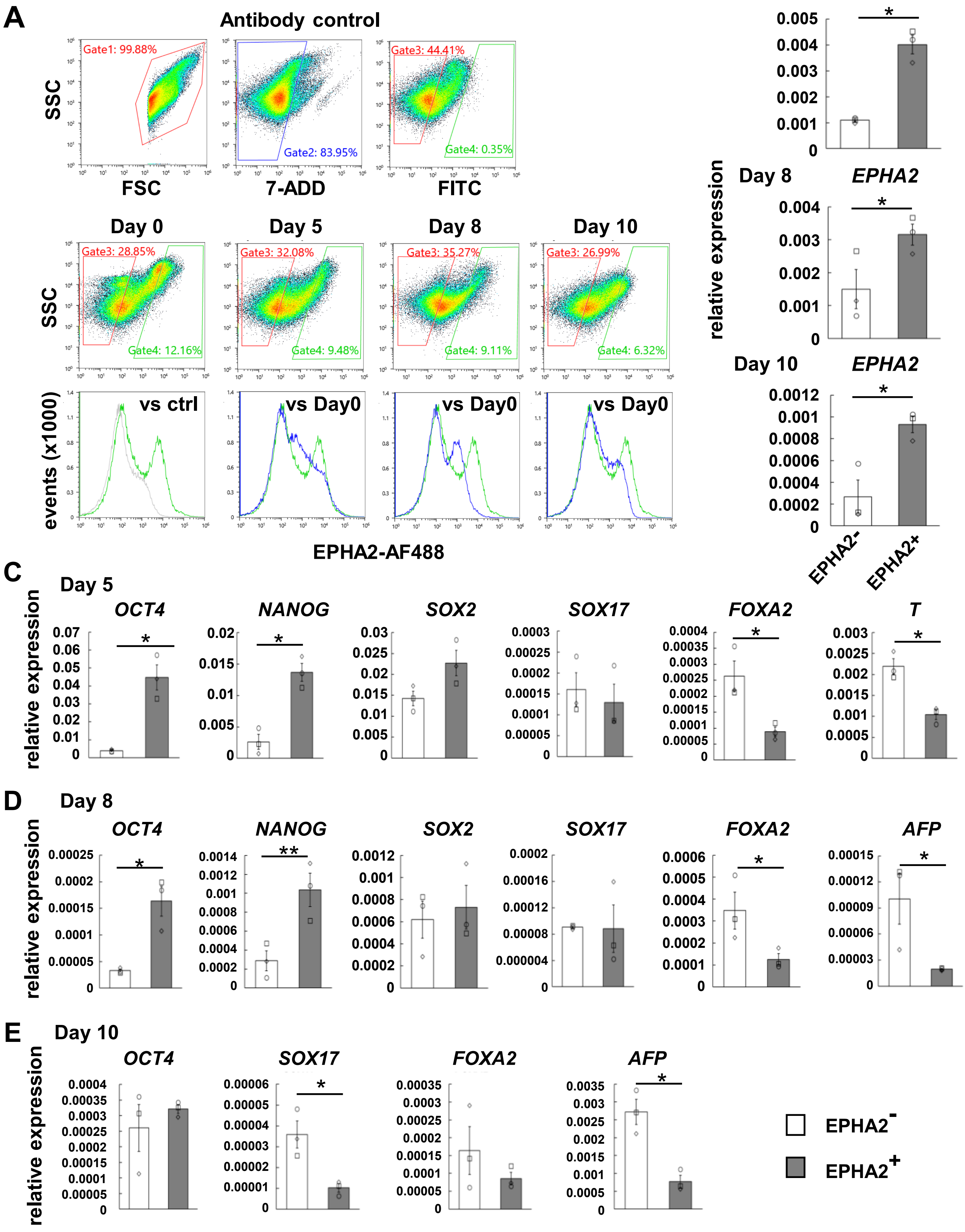

Supplement: szae036_suppl_Supplementary_Figures_S1-S10 [file szae036_suppl_supplementary_figures_s1-s10.zip › Supplementary Figures Intoh 240426/Supplementary Figures Intoh 240426.pdf]
