# Supplementary material for: EPHA2 is a novel cell surface marker of OCT4-positive undifferentiated cells during the differentiation of mouse and human pluripotent stem cells
Source: Stem Cells Transl Med. 2024 May 29;13(8):763–75. doi: 10.1093/stcltm/szae036 (PMC11328934; doi:10.1093/stcltm/szae036)
Supplement: szae036_suppl_Supplementary_Table_1 [file szae036_suppl_supplementary_table_1.docx]

**Supplemental Table 1: Primers and oligo DNA sequences**

Primers for the construction of pOct4-EGFP vector

AseI-mOct4 promotor Fw CGGATTAATAAGCTTTGTGAACTTGGCGGC

NheI-mOct4 promotor Rv CCGCTAGCGGGGAAGGTGGGCACC

Primers for the construction of pMYs-IP-hEPHA2 vector

XhoI-hEPHA2 Fw GCTTCTCGAGATGGAGCTCCAGGCAG

NotI-hEPHA2 Rv GCTGGCGGCCGCTCAGATGGGGATCCCCAC

DNA interference targets Sequence (5'-3')

*mEphA2* Fw GATCCGCGACATGCCTATCTACATGCTGTGAAGCCACAGATGGGCATGTAGATAGGCATGTCGCTTTTTTAT

*mEphA2* Rv CGATAAAAAAGCGACATGCCTATCTACATGCCCATCTGTGGCTTCACAGCATGTAGATAGGCATGTCGCG

control Fw GATCCGTCTTAATCGCGTATAAGGCTAGTGCTCCTGGTTGGCCTTATACGCGATTAAGACTTTTTTAT

control Rv CGATAAAAAAGTCTTAATCGCGTATAAGGCCAACCAGGAGCACTAGCCTTATACGCGATTAAGACG

Mouse genes Sequence (5'-3')

*mEphA2* Fw TGCAAGGTGTCCGATTTTGG

*mEphA2* Rv CGTGGTTTGACAGTTCCCAG

*mBrachyury* (*mT*) Fw CTTTGTTTCTTCCCGCTGAG

*mBrachyury* (*mT*) Rv GCAAACCTGGTCATTCCAGT

*mFgf5* Fw AGGGACGGTCAAGATTCCTT

*mFgf5* Rv AGAACCAGCAGAGTCCCAGA

*mFoxa2* Fw TCAAGGCCTAGAACAGGTC

*mFoxa2* Rv CCACTCAGCCTCTCATTTCC

*mGata4* Fw CACAAGGCTATGCGTCTCC

*mGata4* Rv GCCTCCTTCTTTGCTATCCTC

*mGata6* Fw CAGCAAGATGAATGGCCTCAG

*mGata6* Rv CACGGGACAGTCCAAGCCGCCG

*mKlf4* Fw GTGCCCCGACTAACCGTT

*mKlf4* Rv GTCGTTGAACTCCTCGGTCT

*mMixl1* Fw TGCTACCCGAGTCCAGGAT

*mMixl1* Rv CCTTGAGGATAAGGGCTGAA

*mNanog* Fw CTGTGTTCTCTCAGGCC

*mNanog* Rv GGGATACTCCACTGGTG

*mOct4* Fw TGCGGAGGGATGGCATAC

*mOct4* Rv CTCCAACTTCACGGCATTG

*mPax6* Fw AGTGTCAGTTCCCGTCCAAG

*mPax6* Rv GTGCTTCTAACCGCCATTTC

*mSox1* Fw CCAGGAAAACCCCAAGATGCAC

*mSox1* Rv CGTTAGCCCACCGTTGACAT

*mSox2* Fw GCGGAGTGGAAACTTTTGTCC

*mSox2* Rv CGGGAAGCGTGTACTTATCCTT

*mSox7* Fw TGACCTCTTGCCACCAAGGA

*mSox7* Rv GCCACGGCCACGTATTACAA

*mSox17* Fw AGGGCCGAAGCAGTGTTACACA

*mSox17* Rv TCTCGTGTAGCCCCTCAACTGTTC

*mTwist2* Fw GCAAGATCCAGACGCTCAAG

*mTwist2* Rv TCTTATTGTCCATCTCGTCGC

*mGapdh* Fw ACCCAGAAGACTGTGGATGG

*mGapdh* Rv CACATTGGGGGTAGGAACAC

Human genes Sequence (5'-3')

*hEPHA2* Fw CACATATGAGGACCCCAACC

*hEPHA2* Rv ACACCTCCCCAAACTCTCCT

*hAFP* Fw GTGGTCAGTTTGCAGCATTC

*hAFP* Rv GCAGAGGAGATGTGCTGGAT

*hALB* Fw TGCTGATGAGTCAGCTGAAAA

*hALB* Rv TCAGCCATTTCACCATAGGTT

*hBRACHYURY* (*hT*) Fw AATTGGTCCAGCCTTGGAA

*hBRACHYURY* (*hT*) Rv TGCTCACAGACCACAGGC

*hFOXA2* Fw CATCCGCCACTCGCTCT

*hFOXA2* Rv GAGTCAGGGTGCAGGGTCC

*hGATA4* Fw AGGGCCTCCTTCTTTGCTATC

*hGATA4* Rv ACTCTTGGAACAGCCTGGTC

*hHAND1* Fw TTTGTTCTTCGAATCGTGGTG

*hHAND1* Rv AAAGTGTTTCCCTTGGAACTAA

*hHNF1B* Fw GTGGACCGGATGGTCAGTG

*hHNF1B* Rv GGGTCTTCATAGGGGTGCC

*hMIXL1* Fw GGTACCCCGACATCCACTT

*hMIXL1* Rv TGGAAGGATTTCCCACTCTG

*hNANOG* Fw GATTTGTGGGCCTGAAGAAA

*hNANOG* Rv ATGGAGGAGGGAAGAGGAGA

*hNEUROD1* Fw ATTCTAAGACGCAGAAGCTG

*hNEUROD1* Rv ACTGGTAGGAGTAGGGGTGT

*hOCT4* Fw ACAACAATGAAAATCTTCAGGAGATATGC

*hOCT4* Rv ACAGAACCACACTCGGACCACATCCTTC

*hSOX2* Fw GCACATGAAGGAGCACCCGGATTA

*hSOX2* Rv CGGGCAGCGTGTACTTATCCTTCTT

*hSOX9* Fw GACTACACCGACCACCAGAA

*hSOX9* Rv TCGAGTGAGCTGTGTGTAGA

*hSOX17* Fw TCATGGTGTGGGCTAAGGAC

*hSOX17* Rv TCTGCCTCCTCCACGAAG

*hGAPDH* Fw ACATGGCCTCCAAGGAGTAAG

*hGAPDH* Rv TTGATGGTACATGACAAGGTGCG
